# Steroidal Constituents from Roots and Rhizomes of *Smilacina japonica*

**DOI:** 10.3390/molecules23040798

**Published:** 2018-03-30

**Authors:** Yuwen Cui, Xinjie Yang, Dongdong Zhang, Yuze Li, Li Zhang, Bei Song, Zhenggang Yue, Xiaomei Song, Haifeng Tang

**Affiliations:** 1 Institute of Materia Medica, School of Pharmacy, Fourth Military Medical University, Xi’an 710032, China; polaris_101025@163.com; 2 Department of Pharmacy, Xi’an Medical University, Xi’an 710021, China; 3 Shaanxi Collaborative Innovation Center of Chinese Medicinal Resource Industrialization, Laboratory of New Drugs and Chinese Medicine Foundation Research, Shaanxi Rheumatism and Tumor Center of TCM Engineering Technology Research, School of Pharmacy, Shaanxi University of Chinese Medicine, Xianyang 712046, China; xxx211xxx@126.com (X.Y.); zhangl123666@163.com (L.Z.); liuxingjian1981@163.com (Z.Y.); songxiaom@126.com (X.S.); 4 The College of Pharmacy, Shanghai University of Traditional Chinese Medicine, Shanghai 200001, China; zhangnatprod@163.com; 5 The College of Life Sciences, Northwest University, Xi’an 710069, China; lyz1990yeah@163.com (Y.L.); songbei168@126.com (B.S.)

**Keywords:** *Smilacina japonica*, steroidal constituents, structure identification, cytotoxicity

## Abstract

Four new steroidal constituents (**1**–**4**) along with two known steroidal glycosides (**5** and **6**) were isolated from the roots and rhizomes of *Smilacina japonica*. Analysis of their physicochemical properties and spectroscopic profiles identified the compounds as (25*S*)-5*α*-spirostan-9(11)-en-3β, 17*α*-diol (**1**); (25*S*)-5*α*-spirostan-9(11)-en-3β, 12β-diol (**2**); (25*S*)-5*α*-spirostan-9(11)-en-3β, 17*α*-diol-3-*O*-β-d-glucopyranoside (**3**); (25*S*)-5*α*-spirostan-9(11)-en-3β, 17*α*-diol-3-*O*-β-d-glucopyranosyl-(1→2)-[β-d-glucopyranosyl-(1→3)]-β-d-galactopyranoside (**4**); japonicoside B (**5**); and japonicoside C (**6**). All six compounds showed cytotoxic activity against SMMC-7712, Bel-7402, A549, H460, and K562 human cancer cells.

## 1. Introduction

**Figure 1:**
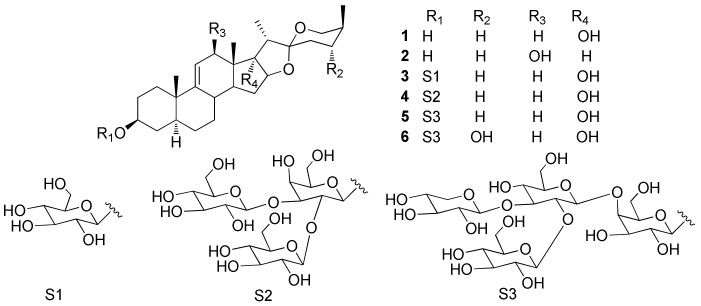
Chemical structures of compounds **1**–**6**.

*Smilacina japonica* A. Gray is a perennial herb of the genus *Smilacina* (Liliaceae) that is mainly distributed in Asia, America, and Europe. As one of about 16 *Smilacina* species in China, *S. japonica* is widely distributed in Hebei, Shanxi, Shaanxi, Gansu, and Henan Provinces and is used in traditional Chinese medicine or consumed as a vegetable. Its roots and rhizomes are known to dispel pathogenic wind, remove dampness, promote blood circulation, and alleviate pain, and have long been used in folk medicine for the treatment of rheumatism, nervous headache, mastitis, carbuncle, and bruises [1,2,3,4,5]. Previous investigations of the bioactive compounds of *S. japonica* revealed four steroidal saponins [6,7,8]. As part of our exploration of the diversity of bioactive compounds in medicinal herbs growing in the Qinba Mountains [9,10,11,12,13,14,15], we investigated the chemical constituents of *S. japonica* in the present study. We identified six steroidal constituents (Figure 1), characterized their structure and pharmacological profiles, and evaluated their cytotoxicity against various human cancer cell lines.

## 2. Results and Discussion

Compound **1** was isolated as a white, amorphous powder and was positive to Liebermann-Burchard and Molisch chemical reactions. The HR-ESI-MS analysis revealed a positive molecular ion peak at *m*/*z* 431.3154 [M + H]^+^, corresponding to the molecular formula C_27_H_42_O_4_ (calculated for 431.3161 [M + H]^+^). The proton ^1^H-NMR spectrum of **1** showed the presence of four methyl protons at *δ*_H_ 0.92 (3H, s, H-18), 0.98 (3H, s, H-19), 1.21 (3H, d, *J* = 7.1 Hz, H-21), and 1.05 (3H, d, *J* = 7.1 Hz, H-27) and an exocyclic olefinic proton at *δ*_H_ 5.51 (1H, d, *J* = 5.3 Hz, H-11). The ^13^C-NMR spectrum showed 27 carbon signals corresponding to one double-bond carbon at *δ*_C_ 145.5 (C-9) and 116.2 (C-11) and four methyl groups at *δ*_C_ 16.4 (C-18), 17.4 (C-19), 8.2 (C-21), and 15.4 (C-27). The quaternary carbon signal at *δ*_C_ 110.5 was identified as an acetal carbon (C-22), which is characteristic of spirostanol saponin. The ^13^C signals of carbons near C-17, C-12 (*δ*_C_ 32.8), C-13 (*δ*_C_ 42.9), C-14 (*δ*_C_ 50.5), C-16 (*δ*_C_ 90.1), and C-21 (*δ*_C_ 8.2) and the further long-range correlation from 21-CH_3_ to the quaternary C-atom at *δ*_C_ 88.9 (C-17) was also observed, suggesting the presence of a hydroxyl group at C-17.

**Figure 2:**
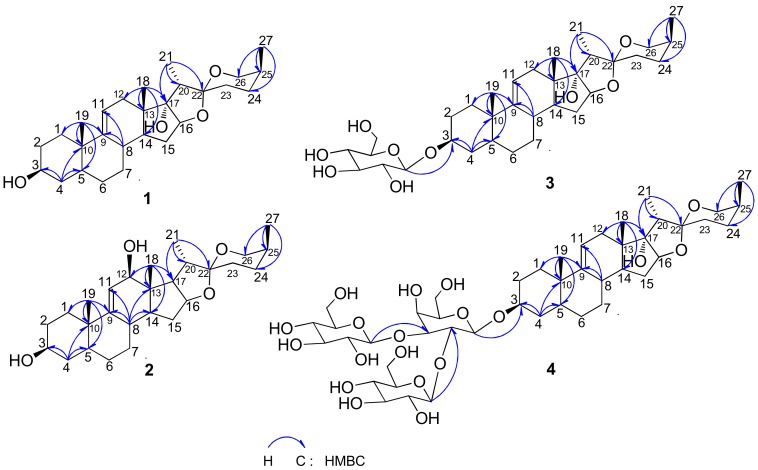
Key HMBC correlations of compounds **1**–**4**.

**Figure 3:**
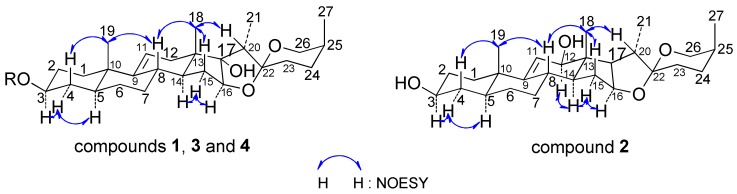
Key NOESY correlations of compounds **1**–**4.**

The above observations were supported by 2D-NMR analysis. The proton and protonated carbon resonances in the NMR spectrum of **1** were unambiguously assigned by the heteronuclear single quantum correlation (HSQC) experiment. Heteronuclear multiple bond correlations (HMBCs) of H-19/C-1, C-5, C-9, and C-10; H-4/C-2 and C-5; H-6/C-5, C-7, and C-8; H-8/C-9, C-11, and C-14; H-18/C-12, C-13, C-14, and C-17; H-15/H-14, H-16, and H-17; H-16/C-15, C-17, and C-22; H-21/C-17, C-20, and C-22; and H-27/C-24, C-25, and C-26 revealed the planar structure of **1** as spirostan-9(11)-en-3,17-diol (Figure 2). Meanwhile, in the nuclear Overhauser effect (NOE) spectrum, NOE correlations between H19/H-4b and H-8, H-4a/H-3, and H-3/H-5 indicated an *α*-axial configuration of H-3 and H-5 and β orientation of H-8, H-19, and 3-OH, with an A/B *trans* ring junction pattern; whereas NOE correlations of H-19/H-8, H-8/H-18, H-18/H-15b, and H-20 and of H-15a/H-14 and H-16 suggested *α*-axial configurations for H-21 and 17-OH (D/E *cis* ring, Figure 3), β orientation for H-18, and B/C and C/D *trans* ring junction patterns. Finally, the absolute configuration of C-25 was deduced as *S* based on the difference in chemical shifts between equatorial (*δ*_H_ 3.24) and axial (*δ*_H_ 4.03) H-26, which is usually Δ*δ* > 0.57 and <0.48 ppm for 25*S* and 25*R* compounds, respectively [16]. Compound **1** was identified as (25*S*)-5*α*-spirostan-9(11)-en-3β, 17*α*-diol (**1**; Figure 1).

Compound **2** was isolated as a white, amorphous powder and was positive to Liebermann-Burchard and Molisch chemical reactions. The HR-ESI-MS spectrum showed a positive molecular ion peak at *m*/*z* 431.3152 [M + H]^+^ corresponding to a molecular formula of C_27_H_42_O_4_ (calculated for 431.3161 [M + H]^+^). The ^1^H-NMR spectrum of **2** showed the presence of four methyl protons at *δ*_H_ 1.09 (3H, s, H-18), 0.99 (3H, s, H-19), 1.42 (3H, d, *J* = 6.9 Hz, H-21), and 1.08 (3H, d, *J* = 6.9 Hz, H-27) and an exocyclic olefinic proton at *δ*_H_ 5.59 (1H, broad singlet, H-11). The ^13^C-NMR spectrum showed 27 carbon signals—including one double-bond carbon at *δ*_C_ 148.5 (C-9) and 123.9 (C-11); four methyl groups at *δ*_C_ 10.9 (C-18), 18.0 (C-19), 14.0 (C-21), and 16.3 (C-27); and an quaternary carbon signal at *δ*_C_ 110.1 that was identified as an acetal carbon (C-22). A comparison of proton and carbon data showed that the resonances of **2** were similar to those of **1** (Table 1), except for the absence of 17-OH and appearance of 12-OH in the former. Moreover, the down-field shifted methylene signal at *δ*_C_ 123.9 (C-11) and the up-field Me-18 signal at *δ*_C_ 10.9 suggested the existence of one β-oriented hydroxyl group at C-12 (*δ*_C_ 78.6), which was further confirmed by ROESY correlations between H-12 and H-14, H-14 and H-15, H-15 and H-16. The chemical structure of **2** was supported by 2D-NMR data from HSQC, HMBC (Figure 2), and NOE spectroscopy (NOESY) (Figure 3) experiments. Compound **2** was identified as (25*S*)-5*α*-spirostan-9(11)-en-3β, 12β-diol (**2**; Figure 1).

Compound **3** was isolated as a white, amorphous powder and was positive to Liebermann-Burchard and Molisch chemical reactions. The HR-ESI-MS spectrum showed a negative molecular ion peak at *m*/*z* 591.3508 [M + H]^−^ corresponding to a molecular formula of C_33_H_52_O_9_ (calculated for 591.3533 [M + H]^−^). The ^1^H-NMR spectrum of **3** revealed the presence of four methyl protons at *δ*_H_ 0.92 (3H, s, H-18), 0.68 (3H, s, H-19), 1.22 (3H, d, *J* = 7.1 Hz, H-21), and 1.05 (3H, d, *J* = 7.1 Hz, H-27) and an anomeric proton at *δ*_H_ 4.98 (1H, d, *J* = 7.6 Hz, H-Glc-1). The ^13^C-NMR spectrum showed 33 carbon signals; one double-bond carbon at *δ*_C_ 145.5 (C-9) and 116.4 (C-11) and four methyl groups at *δ*_C_ 16.6 (C-18), 17.4 (C-19), 8.5 (C-21), and 15.6 (C-27), as well as an anomeric carbon at *δ*_C_ 102.2 (C-Glc-1). In addition, the quaternary carbon signal at *δ*_C_ 109.8 was identified as an acetal carbon (C-22), which is characteristic of spirostanol saponin. A comparison of the proton and carbon data with those of compound **1** (Table 1) indicated that a glucose unit (102.2, 72.1, 76.4, 69.8, 74.9, and 62.0) was linked to the C-3 of the aglycone (C_3_ + 6.8 ppm). Acid hydrolysis of **3** yielded D-glucose, which was confirmed by GC analysis of the trimethylsilyl-l-cysteine derivatives of compound **3** hydrolysate and authentic sugars. The large *J* values (*J* >7.0 Hz) of the anomeric proton signals reflected the β configuration of D-glucose. Compound **3** was identified as (25*S*)-5*α*-spirostan-9(11)-en-3β, 17*α*-diol 3-*O*-β-d-glucopyranoside (**3**; Figure 1) based on HSQC, HMBC (Figure 2), and NOESY (Figure 3) data.

Compound **4** was isolated as a white, amorphous powder and was positive to Liebermann-Burchard and Molisch chemical reactions. The HR-ESI-MS spectrum showed a negative molecular ion peak at *m*/*z* 915.4565 [M + H]^−^, corresponding to a molecular formula of C_45_H_72_O_19_ (calculated for 915.4590 [M + H]^−^). NMR spectra for **4** and **1** showed similar features, except that the 3-OH in **1** was replaced by β-d-glucopyranosyl-(1→2)-[β-d-glucopyranosyl-(1→3)]-β-d-galactopyranoside in **4.** The HMBC spectrum of **4** (Figure 2) revealed correlations between H-Glc-1′′/C-Gal-3, H-Glc-1′/C-Gal-2, and H-Gal-1/C-3, with the two glucose units linked to C-Gal-2 and C-Gal-3 of the inner galactose unit, which was itself linked to C-3 of aglycone. This was confirmed by acid hydrolysis and subsequent GC analysis of the hydrolysates and 2D-NMR analysis. Coupling constants of the anomeric proton signals suggested β-configuration of d-glucose and d-galactose. Based on this evidence, compound **4** was identified as (25*S*)-5*α*-spirostan-9(11)-en-3β, 17*α*-diol 3-*O*-β-d-glucopyranosyl-(1→2)-[β-d-glucopyranosyl-(1→3)]-β-d-galactopyranoside (**4**; Figure 1).

The two known glycosides were identified as *japonicoside* B (**5**, Figure 1) and *japonicoside* C (**6**, Figure 1); (25*S*)-5*α*-spirostan-9(11)-en-3β, 17*α*-diol 3-*O*-β-d-glucopyranosyl-(1→2)-[β-d-xylopyranosyl-(1→3)]-β-d-glucopyranosyl(1→4)-β-d-galactopyranoside (**5**), (25*S*)-5*α*-spirostan-9(11)-en-3β, 17*α*, 24*α*-diol 3-*O*-β-d-glucopyranosyl-(1→2)-[β-d-xylopyranosyl-(1→3)]-β-d-glucopyranosyl(1→4)-β-d-galactopyranoside (**6**), respectively, by comparing their spectroscopic data (Supplementary Materials) with those reported in the literature [6].

Because steroidal saponins have been reported to possess varying cytotoxic activity against various cancer cell lines [17,18,19], in this paper, the cytotoxic activity against human SMMC-7712, Bel-7402, A549, H460, and K562 tumor cells of compounds **1**–**6** were evaluated by MTT method and all the compounds exhibited cytotoxicity with the cell lines. Compound **2** exhibited a more potent antitumor effect than **1**: it seemed that the presence of a free hydroxyl group at C-12 was more potent than a free hydroxyl group at C-17. Compounds **1**, **3**–**5** shared the same aglycone, but exhibited different activities. This suggested that the structural differences such as the category, the number, and the sequence of the oligosaccharide chain at C-3 played a role in terms of antitumor effect. Meanwhile, compared with **5**, compound **6** has one more free hydroxyl group than **5**, but their cytotoxic activity have no differences. It seemed that the presence of a free hydroxyl group at C-24 has less effect on the cytotoxic activity. Furthermore, compounds **2** and **4** exhibited moderate cytotoxicity against A549 cells with IC_50_ values of 14.4 μM and 12.3 μM, respectively, while compounds **1**, **3**, **5** and **6** displayed no activity (IC_50_ > 100 μM). It seemed that the differences of the composition of the oligosaccharide chain have an effect on the cytotoxic activity and selectivity of the various cell lines. Thus, our results indicated that the antitumor effects of steroidal constituents from this species are very sensitive to their precise functionalization. Therefore, more extensive studies are needed before a clear structure-activity relationship can be reached.

## 3. Materials and Methods

### 3.1. Materials

Optical rotations were recorded in MeOH using a Rudolph research analytical automatic polarimeter (Rudolph Research Analytical, Hackettstown, NJ, USA). Infrared (IR) spectra were recorded on a TENSOR-27 instrument (Bruker, Billerica, MA, USA). High-resolution electrospray ionization mass spectrometry (HR-ESI-MS) analysis was carried out on a model 6550 quadrupole time-of-flight mass spectrometer (Agilent Technologies, Santa Clara, CA, USA). One-dimensional (1D) and 2D nuclear magnetic resonance (NMR) spectra were recorded on an AVANCE400 instrument (Bruker) with tetramethylsilane as an internal standard. High-performance liquid chromatography (HPLC) was performed on a 2695 Separations Module (Waters, Milford, MA, USA) coupled with a 2996 Photodiode Array Detector and octadecylsilyl (ODS)-3 column (4.6 × 250 mm, 5 mm particles; COSMOSIL, Tokyo, Japan). Semi-preparative HPLC was performed on a LC-6AD pump equipped with a SPD-20A ultraviolet detector and an Ultimate XB-C18 column (10 × 250 mm, 5 mm particles) (Shimadzu, Kyoto, Japan). Gas chromatography (GC) was performed on an 7890 A chromatograph (Agilent Technologies, Santa Clara, CA, USA) equipped with an HP-5 capillary column (30 m × 320 mm × 0.25 mm; Agilent Technologies). Sephadex LH-20 and C-18 (40–75 mm) silica gels were purchased from GE Healthcare Bio-Sciences AB (Uppsala, Sweden), and silica gel was also purchased from Qingdao Haiyang Chemical Group Corporation (Qingdao, China).

### 3.2. Plant Material

*Smilacina japonica* A. Gray was collected in August of 2014 from the Taibai region of the Qinba Mountains in the Shaanxi Province of China and was authenticated by Professor Jitao Wang (Shaanxi University of Chinese Medicine). A voucher specimen (herbarium no. 20140826) has been deposited in the Medicinal Plants Herbarium, Shaanxi University of Chinese Medicine (Xianyang, China).

### 3.3. Extraction and Isolation

The air-dried powder of *S. japonica* rhizomes and roots of (6.3 kg) was extracted three times with 80% EtOH under reflux at 80 °C. After removing the solvent, the concentrated residue was successively partitioned with petroleum ether and *n*-BuOH. The *n*-BuOH extract (200 g) was subjected to column chromatography (CC) on silica gel with gradient elution (CHCl_3_–MeOH–H_2_O, 100:0:0−65:35:10), which yielded five fractions (Fr.1–5). Fr.1 (10.5 g) was subjected to CC on silica gel; elution with CHCl_3_–MeOH–H_2_O (100:0:0–10:1:0.1) yielded compound **1** (8.3 mg) and compound **2** (6.8 mg). Fr.4 (22 g) was subjected to CC on silica gel; elution with CHCl_3_–MeOH–H_2_O (100:0:0–70:30:5) yielded four subfractions (Fr.4-1–4-4). Fr.4-1 (1.6 g) was subjected to CC on ODS gel; elution with MeOH-H_2_O (10:90–70:30) yielded compound **3** (15.5 mg). Fr.4-3 (3.4 g) was subjected to CC on ODS gel; elution with MeOH–H_2_O (10:90–70:30) yielded three subfractions (Fr.4-3-1–4-3-3). Fr.4-3-2 (188 mg) was purified by HPLC (flow rate: 1.5 mL/min) with MeCN–H_2_O (46:54) as the mobile phase, yielding compound **4** (18.6 mg; retention time [*t*_R_] = 41.2 min), compound **5** (37.6 mg; *t*_R_ = 33.4 min), and compound **6** (11.6 mg; *t*_R_ = 31.2 min).

### 3.4. (25S)-5α-Spirostan-9(11)-en-3β,17α-diol (Compound ***1***)

This compound was a white amorphous powder (purity >98%) with the following spectral features. ${\left[\alpha \right]}_{D}^{23.1}$ −31.5 (*c* 1.57, MeOH); IR (KBr)ν_max_: 3407, 2934, 1653, 1072, 1035, 979, 913, 892, 845 cm^−1^; ^1^H- and ^13^C-NMR spectral data (Table 1): positive HR-ESI-MS *m*/*z* 431.3154 [M + H]^+^ (calculated for C_27_H_43_O_4_: 431.3161 [M + H]^+^).

### 3.5. (25S)-5α-Spirostan-9(11)-en-3β,12β-diol (Compound ***2***)

This compound was a white amorphous powder (purity of 94%) with the following spectral features. ${\left[\alpha \right]}_{D}^{23.3}$ −39.0 (*c* 0.61, MeOH); IR (KBr)ν_max_: 3407, 2929, 1664, 1070, 1037, 979, 915, 893, 847 cm^−1^; ^1^H- and ^13^C-NMR spectral data (Table 1): positive HR-ESI-MS *m*/*z* 431.3152 [M + H]^+^ (calculated for C_27_H_43_O_4_: 431.3161 [M + H]^+^).

### 3.6. (25S)-5α-Spirostan-9(11)-en-3β,17α-diol 3-O-β-d-glucopyranoside (Compound ***3***)

This compound was a white amorphous powder (purity >98%) with the following spectral features. ${\left[\alpha \right]}_{D}^{22.9}$ −96.6 (*c* 0.16, MeOH); IR (KBr)ν_max_: 3410, 2939, 1658, 1071, 1037, 978, 916, 893, 845 cm^−1^; ^1^H- and ^13^C-NMR spectral data (Table 1): negative HR-ESI-MS *m*/*z* 591.3508 [M − H]^−^ (calculated for C_33_H_51_O_9_: 591.3533 [M − H]^−^).

### 3.7. (25S)-5α-Spirostan-9(11)-en-3β,17α-diol3-O-β-d-glucopyranosyl-(1→2)-[β-d-glucopyranosyl-(1→3)]-β-d-galactopyranoside (Compound ***4***)

This compound was a white amorphous powder (purity >98%) with the following spectral features. ${\left[\alpha \right]}_{D}^{23.0}$ −21.3 (*c* 0.70, MeOH); IR (KBr)ν_max_: 3410, 2930, 1652, 1072, 1035, 977, 915, 893, 847 cm^−1^; ^1^H- and ^13^C-NMR spectral data (Table 1): negative HR-ESI-MS *m*/*z* 915.4565 [M − H]^−^ (calculated for C_45_H_71_O_19_: 915.4590 [M − H]^−^).

### 3.8. Acid Hydrolysis of Compounds ***3***–***4*** and Determination of Absolute Configuration of Sugars

Compounds **3** and **4** (3 and 4 mg) were individually hydrolyzed with 1 N HCl–dioxane (1:1, 3 mL) at 60 °C for 6 h. After dilution with H_2_O (5 mL), the reaction mixture was extracted with EtOAc, yielding distinct EtOAc and H_2_O phases. The latter was evaporated under reduced pressure. After adding H_2_O (5 mL), the acidic solution was evaporated again; this procedure was repeated until a neutral solution was obtained. This was then evaporated and dried in a vacuum, yielding a monosaccharide residue that was dissolved in pyridine (0.5 mL); 2 mg of l-cysteine methyl ester hydrochloride was added and the mixture was maintained at 60 °C for 2 h, evaporated under a stream of N_2_, and dried in a vacuum. After adding 0.2 mL of *N*-trimethylsilylimidazole, the mixture was maintained at 60 °C for 1 h and then partitioned between n-hexane and H_2_O (2 mL each). The n-hexane extract was analyzed by GC under the following conditions: HP-5 capillary column (30 m × 320 mm × 0.25 μm); flame ionization detector; detector temperature = 280 °C; injection temperature = 250 °C; initial temperature = 100 °C for 2 min, followed by an increase to 280 °C at a rate of 10 °C/min; final temperature = 280 °C for 5 min; and N_2_ gas as a carrier. The absolute configurations of sugars isolated from the hydrolysates of compounds **3** and **4** were determined by comparing the *t*_R_ of their trimethylsilyl-l-cysteine derivatives d-glucose (*t*_R_ = 21.22 min) and D-galactose (*t*_R_ = 21.56 min) with those of authentic sugars prepared by a similar procedure.

### 3.9. Cytotoxicity Assay

The cytotoxic activity of the isolated compounds against SMMC-7712, Bel-7402, A549, H460, and K562 human cancer cell lines was evaluated with the (3-(4,5-dimethylthiazol-2-yl)-2, 5-diphenyltetrazolium bromide) assay using 5-fluorouracil as a positive control. Briefly, 1 × 10^4^ cells mL^−1^ were seeded in 96-well plates and allowed to adhere for 24 h. Compounds **1**−**6** were dissolved in dimethylsulfoxide (DMSO) and 6-fold dilutions were prepared in complete medium (from 100–0.1 μmol L^−1^) for determination of inhibition rate. After incubation at 37.8 °C for 24 h, the supernatant was removed and DMSO (100 μL) was added to each well. The inhibition rate and half-maximal inhibitory concentration (IC_50_) were calculated. Compounds **1**−**6** showed cytotoxicity against human SMMC-7712, Bel-7402, A549, H460, and K562 cell lines; the IC_50_ values are shown in Table 2.

## Figures and Tables

**Table 1:** ^1^H-NMR and ^13^C-NMR data for compounds **1**–**4** ^†^.

	1		2		3		4	
No.	δH	δC	δH	δC	δH	δC	δH	δC
1	1.44, 1H, ca.	35.3	1.46, 1H, ca.	36.1	1.26, 1H, ca.	35.3	1.25, 1H, ca.	35.3
	1.75, 1H, ca.		1.66, 1H, ca.		1.61, 1H, ca.		1.61, 1H, ca.	
2	1.73, 1H, ca.	31.7	1.67, 1H, ca.	32.9	1.73, 1H, ca.	29.5	1.73, 1H, ca.	29.5
	2.06, 1H, ca.		2.19, 1H, ca.		2.06, 1H, ca.		2.06, 1H, ca.	
3	3.84, 1H, ca.	69.6	3.86, 1H, ca.	70.4	3.97, 1H, ca.	76.4	3.94, 1H, ca.	76.7
4	1.51, 1H, ca.	34.4	1.53, 1H, ca.	39.3	1.36, 1H, ca.	34.4	1.36, 1H, ca.	34.4
	1.76, 1H, ca.		1.84, 1H, ca.		1.87, 1H, ca.		1.88, 1H, ca.	
5	1.25, 1H, ca.	42.9	1.23, 1H, ca.	43.7	1.03, 1H, ca.	42.6	1.06, 1H, ca.	42.7
6	1.22, 1H, ca.	28.1	1.24, 1H, ca.	28.9	1.16, 1H, ca.	28.2	1.18, 1H, ca.	28.2
	1.38, 1H, ca.		1.28, 1H, ca.		1.25, 1H, ca.		1.24, 1H, ca.	
7	0.95, 1H, ca.	33	0.94, 1H, ca.	33.5	0.92, 1H, ca.	33	0.93, 1H, ca.	33
	1.75, 1H, ca.		1.81, 1H, ca.		1.73, 1H, ca.		1.73, 1H, ca.	
8	2.24, 1H, ca.	36.1	2.18, 1H, ca.	36.3	2.09, 1H, ca.	36.2	2.08, 1H, ca.	36.2
9	−	145.5	−	148.5	−	145.5	−	145.5
10	−	37.5	−	38.2	−	37.6	−	37.6
11	5.51, 1H, d, *J* = 5.3 Hz	116.2	5.59, 1H, brs	123.9	5.45, 1H, d, *J* = 5.3 Hz	116.4	5.45, 1H, d, *J* = 5.3 Hz	116.4
12	1.78, 1H, ca.	32.8	4.33, 1H, brs	78.6	1.77, 1H, ca.	33	1.78, 1H, ca.	33
	3.07, 1H, d, *J* = 17.4 Hz				3.06, 1H, d, *J* = 17.4 Hz		3.06, 1H, d, *J* = 17.4 Hz	
13	−	42.9	−	45.2	−	43.1	−	43.1
14	2.13, 1H, ca.	50.5	1.48, 1H, ca.	53.2	2.13, 1H, ca.	50.6	2.13, 1H, ca.	50.6
15	1.51, 1H, ca.	31.9	1.52, 1H, ca.	32.4	1.53, 1H, ca.	32.1	1.52, 1H, ca.	32.1
	2.32, 1H, ca.		2.07, 1H, ca.		2.32, 1H, ca.		2.31, 1H, ca.	
16	4.45, 1H, d, *J* = 7.2 Hz	90.1	4.62, 1H, ca.	81.5	4.48, 1H, d, *J* = 7.2 Hz	90.3	4.45, 1H, d, *J* = 7.2 Hz	90.3
17	−	88.9	2.34, 1H, t, *J* = 6.0 Hz	61.9	−	89.1	−	89.1
18	0.92, 3H, s	16.4	0.1.09, 3H, s	10.9	0.92, 3H, s	16.6	0.92, 3H, s	16.6
19	0.98, 3H, s	17.4	0.99, 3H, s	18.0	0.68, 3H, s	17.4	0.84, 3H, s	17.4
20	2.23, 1H, ca.	44.8	2.14, 1H, ca.	43.8	2.21, 1H, ca.	45.1	2.22, 1H, ca.	45.1
21	1.21, 3H, d, *J* = 7.1 Hz	8.2	1.42, 3H, d, *J* = 6.9 Hz	14.0	1.23, 3H, d, *J* = 7.1 Hz	8.5	1.22, 3H, d, *J* = 7.1 Hz	8.5
22	−	110.5	−	110.1	−	109.8	−	109.8
23	1.45, 1H, ca.	25.6	1.47, 1H, ca.	26.4	1.46, 1H, ca.	25.8	1.46, 1H, ca.	25.8
	1.92, 1H, ca.		1.92, 1H, ca.		1.92, 1H, ca.		1.91, 1H, ca.	
24	1.32, 1H, ca.	24.8	1.34, 1H, ca.	26.2	1.33, 1H, ca.	25.1	1.32, 1H, ca.	25.1
	2.06, 1H, ca.		2.04, 1H, ca.		2.05, 1H, ca.		2.03, 1H, ca.	
25	1.55, 1H, ca.	26.5	1.59, 1H, ca.	27.6	1.55, 1H, ca.	26.3	1.54, 1H, ca.	26.7
26	3.24, 1H, d, *J* = 11.0 Hz	64.2	3.33, 1H, d, *J* = 11.0 Hz	65.2	3.24, 1H, d, *J* = 11.0 Hz	64.4	3.24, 1H, d, *J* = 11.0 Hz	64.4
	4.04, 1H, dd, *J* = 2.4, 11.0 Hz		4.09, 1H, dd, *J* = 2.4, 11.0 Hz		4.03, 1H, dd, *J* = 2.4, 11.0 Hz		4.04, 1H, dd, *J* = 2.4, 11.0 Hz	
27	1.05, 3H, d, *J* = 7.1 Hz	15.4	1.08, 3H, d, *J* = 6.9 Hz	16.3	1.05, 3H, d, *J* = 7.1 Hz	15.6	1.06, 3H, d, *J* = 7.1 Hz	15.6
Sugar								
Gal-1					4.98, 1H, d, *J* = 7.6 Hz	102.2	4.91, 1H, d, *J* = 7.6 Hz	101.8
2					4.47, 1H, ca.	72.1	4.62, 1H, ca.	80.5
3					4.18, 1H, t, *J* = 6.0 Hz	76.4	4.18, 1H, ca.	85.5
4					4.61, 1H, d, *J* = 3.0 Hz	69.8	4.53, 1H, ca.	72.7
5					4.24, 1H, dd, *J* = 3.0, 9.4 Hz	74.9	4.01, 1H, ca.	77.6
6					4.48, 2H, ca.	62	4.26, 1H, ca.	59.9
							4.80, 1H, ca.	
Glc-1′							5.16, 1H, d, *J* = 7.5 Hz	104.6
2′							4.13, 1H, ca.	75
3′							3.81, 1H, ca.	78.3
4′							3.99, 1H, ca.	71.2
5′							4.07, 1H, ca.	76.2
6′							4.02, 1H, ca.	60.9
							4.65, 1H, ca.	
Glc-1′′							5.27, 1H, d, *J* = 7.5 Hz	106.4
2′′							4.06, 1H, ca.	74.5
3′′							3.97, 1H, ca.	77.9
4′′							4.26, 1H, ca.	69.6
5′′							4.12, 1H, ca.	77
6′′							4.37, 1H, ca.	62.6
							4.59, 1H, ca.	

^†^ Assignments aided by the HSQC, HMBC, and NOESY experiments; ^1^H- and ^13^C-NMR were measured at 400 and 100 MHz in pyridine-*d*_5_.

**Table 2:** Cytotoxicities of compounds **1**–**6** against five human cancer cell lines in vitro (IC_50_, μM) ^a^.

Compounds	Cell Lines				
	SMMC-7721	Bel-7402	A549	H460	K562
^b^ 5-Fu	2.4 ± 1.9	4.3 ± 2.1	4.0 ± 1.6	1.7 ± 2.8	1.0 ± 0.9
1	10.4 ± 3.9	13.3 ± 4.6	>100	33.7 ± 2.2	25.5 ± 3.5
2	11.2 ± 4.4	6.9 ± 1.7	14.4 ± 2.8	26.2 ± 2.7	29.3 ± 5.3
3	8.6 ± 3.4	11.4 ± 4.1	>100	31.4 ± 1.7	24.2 ± 2.9
4	7.2 ± 4.6	4.4 ± 1.1	12.3 ± 2.5	21.8 ± 2.1	>100
5	7.4 ± 5.8	31.9 ± 2.1	>100	34.4 ± 3.2	15.8 ± 5.4
6	8.8 ± 5.3	30.3 ± 2.4	>100	30.3 ± 3.7	12.3 ± 5.5

^a^ IC_50_ values are means from three independent experiments (average ± SD) in which each compound concentration was tested in three replicate wells; ^b^ 5-fluorouracil (5-Fu) as positive control.

## Supplementary Materials

The physical and spectroscopic data of compounds **5**–**6**, and NMR and MS spectra of compounds **1**–**4** are available online.
