# Supplementary material for: Steroidal Constituents from Roots and Rhizomes of Smilacina japonica
Source: Molecules. 2018 Mar 30;23(4):798. doi: 10.3390/molecules23040798 (PMC6017528; doi:10.3390/molecules23040798)

Supporting Materials

Steroidal Constituents from Roots and Rhizomes of *Smilacina japonica*

Yuwen Cui[1, 2,+] , Xinjie Yang[3,+], Dongdong Zhang [4] , Yuze Li [5], Li Zhang[3], Bei Song[5], Zhenggang Yue[3], Xiaomei Song[3], Haifeng Tang [1,*]

[1] Institute of Materia Medica, School of Pharmacy, Fourth Military Medical University, Xi'an 710032, PR China; polaris_101025@163.com (Y.-W.C.); tanghaifeng71@163.com (H.-F.T.)

[2] Department of Pharmacy, Xi'an Medical University, Xi'an 710021, PR China; polaris_101025@163.com (Y.-W.C.)

[3] Shaanxi Collaborative Innovation Center of Chinese Medicinal Resource Industrialization, Shaanxi Province Key Laboratory of New Drugs and Chinese Medicine Foundation Research, Shaanxi Rheumatism and Tumor Center of TCM Engineering Technology Research, School of Pharmacy, Shaanxi University of Chinese Medicine, Xianyang 712046, PR China; xxx211xxx@126.com(X.-J.Y.); zhangl123666@163.com(L.Z); liuxingjian1981@163.com(Z.-G.Y); songxiaom@126.com(X.-M.S)

[4] The College of Pharmacy, Shanghai University of Traditional Chinese Medicine ; Shanghai 200001, PRChina; zhangnatprod@163.com(D.-D.Z)

[5] The College of Life Sciences，Northwest University; Xi'an 710069, PRChina; songbei168@126.com (B.S.), lyz1990yeah@163.com(Y.-Z.L)

* Correspondence: tanghaifeng71@163.com (H.-F.T.); Tel.: +86-29-8477-4748 (H.-F.T.)

† The authors contribute equally to this work.

Contents

Physical and spectroscopic data of compounds **5–6**

NMR (1D and 2D) and MS spectra of compounds 1–4

S1. The IR spectrum of compound 1 (in KBr)

S2. The HR-ESI-MS spectrum of compound 1 (in MeOH)

S3. The ^{1}H NMR spectrum of compound 1 (in pyridine-d5)

S4. The ^{13}C NMR spectrum of compound 1 (in pyridine-d5)

S5. The HSQC spectrum of compound 1 (in pyridine-d5)

S6. The HMBC spectrum of compound 1 (in pyridine-d5)

S7. The NOESY spectrum of compound 1(in pyridine-d5)

S8. The IR spectrum of compound 2 (in KBr)

S9. The HR-ESI-MS spectrum of compound 2 (in MeOH)

S10. The ^{1}H NMR spectrum of compound 2 (in pyridine-d5)

S11. The ^{13}C NMR spectrum of compound 2 (in pyridine-d5)

S12. The HSQC spectrum of compound 2 (in pyridine-d5)

S13. The HMBC spectrum of compound 2 (in pyridine-d5)

S14. The NOESY spectrum of compound 2 (in pyridine-d5)

S15. The IR spectrum of compound 3 (in KBr)

S16. The HR-ESI-MS spectrum of compound 3 (in MeOH)

S17. The ^{1}H NMR spectrum of compound 3 (in pyridine-d5)

S18. The ^{13}C NMR spectrum of compound 3 (in pyridine-d5)

S19. The HSQC spectrum of compound 3 (in pyridine-d5)

S20. The HMBC spectrum of compound 3 (in pyridine-d5)

S21. The NOESY spectrum of compound 3 (in pyridine-d5)

S22. The IR spectrum of compound 4 (in KBr)

S23. The HR-ESI-MS spectrum of compound 4 (in MeOH)

S24. The ^{1}H NMR spectrum of compound 4(in pyridine-d5)

S25. The ^{13}C NMR spectrum of compound 4 (in pyridine-d5)

S26. The HSQC spectrum of compound 4 (in pyridine-d5)

S27. The HMBC spectrum of compound 4 (in pyridine-d5)

S28. The NOESY spectrum of compound 4 (in pyridine-d5)

Physical and spectroscopic data of compounds 5–6

Compound **5**: White amorphous powder; was positive to Liebermann-Burchard and Molisch chemical reactions. ESI-MS m/z 1047.5 ([M_H]—)，1H-NMR (400MHz，pyridin-d5)，5.43（1H，*d*，*J* = 4.3Hz，H-11），0.83（3H，*s*，CH_3-18），0.91（3H，*s*，CH_3-19, 25S），0.93（3H，*s*，CH_3-19, 25R），0.92 (3H，*d*，*J* = 7.0 Hz)，1.07（3H，*d*，*J* = 7.1Hz，CH_3-27），4.98（1H，d ，*J* = 7.6Hz，H-Gal-1），5.19（1H，d ，J = 7.9Hz，H-Glc′-1），5.26（1H，d ，J = 7.7Hz，H-Xyl-1），5.59（1H，d ，J = 7.3Hz，H-Glc″-1）; 13C-NMR (100MHz，pyridin-d5)，35.5（C-1），29.3（C-2），78.2（C-3），34.1（C-4），43.8（C-5），29.3 (C-6)，33.8（C-7），36.9（C-8），146.6（C-9），38.5（C-10），117.5（C-11），34.1（C-12），43.8（C-13），51.7（C-14），31.3（C-15），25S: 91.4 (C-16)，90.2（C-17），16.7（C-18），18.5（C-19），45.8（C-20），9.5（C-21），110.9（C-22），26.5（C-23），25.7（C-24），27.4（C-25），65.1（C-26），17.2（C-27）; 25R: 89.9 (C-16)，89.8（C-17），16.7（C-18），18.5（C-19），45.7（C-20），9.1（C-21），109.9（C-22），31.7（C-23），29.1（C-24），30.5（C-25），66.8（C-26），17.2（C-27）;103.0（Gal-1），75.6（Gal-2），73.7（Gal-3），79.2（Gal-4），76.7（Gal-5），61.1（Gal-6）; 105.3（Glc-1′），81.8（Glc-2′），87.3（Glc-3′），71.5（Glc-4′），77.9（Glc-5′），63.4（Glc-6′）; 105.4（Glc-1″），75.5（Glc-1″），78.2（Glc-1″），71.2（Glc-4″），78.1（Glc-5″），62.9（Glc-6″）; 105.7（Xyl-1），75.5（Xyl-1），79.1（Xyl-3），70.9（Xyl-4），67.8（Xyl-5）.Compared to the physical and spectroscopic data with literature values, compound **5** was identified as (25*S*)-5*α*-spirostan-9(11)-en-3*β*, 17*α*-diol 3-O-*β*-*D*-glucopyranosyl-(1→2)-[*β*-*D*- xylopy ranosyl --(1→3)]-*β*-*D*-glucopyranosyl(1→4)-*β*-*D*-galactopyranoside(japonicoside B).

Compound **6**: White amorphous powder; was positive to Liebermann-Burchard and Molisch chemical reactions. ESI-MS *m*/*z* 1063.5 ([M-H]$^{-}$), ^{1}H-NMR (400MHz，pyridin-d_5) , 5.43（1H，d，*J* = 4.3Hz，H-11），0.83（3H，s，CH_3-18），1.22（3H，s，CH_3-19），0.92 (3H，d，*J* = 7.0 Hz)，1.07（3H，d，*J* = 7.1Hz，CH_3-27），4.98（1H，

d ，J = 7.6Hz，H-Gal-1），5.19（1H，d ，J = 7.9Hz，H-Glc′-1），5.26（1H，d ，J = 7.7Hz，H-Xyl-1），5.59（1H，d ，J = 7.3Hz，H-Glc″-1）；^{13}C-NMR (100MHz，pyridin-d_5), 35.5（C-1），29.3（C-2），78.2（C-3），34.1（C-4），43.8（C-5），29.3 (C-6)，33.3（C-7），36.4（C-8），146.6（C-9），37.3（C-10），117.5（C-11），32.5（C-12），43.8（C-13），51.7（C-14），31.3（C-15），91.4 (C-16)，90.2（C-17），16.7（C-18），18.5（C-19），45.3（C-20），9.5（C-21），110.9（C-22），36.4（C-23），65.5（C-24），34.1（C-25），63.5（C-26），9.8（C-27），103.0（Gal-1），75.6（Gal-2），73.7（Gal-3），79.2（Gal-4），76.7（Gal-5），61.1（Gal-6）；105.3（Glc-1′），81.8（Glc-2′），87.3（Glc-3′），71.5（Glc-4′），77.9（Glc-5′），63.4（Glc-6′）；105.4（Glc-1″），75.5（Glc-1″），78.2（Glc-1″），71.2（Glc-4″），78.1（Glc-5″），62.9（Glc-6″）；105.7（Xyl-1），75.5（Xyl-1），79.1（Xyl-3），70.9（Xyl-4），67.8（Xyl-5）。Compared to the physical and spectroscopic data with literature values, compound **6** was identified as (25*S*)-5*α*-spirostan-9(11)-en-3*β*, 17*α*, ,24*α*-diol 3-O-*β*-*D*-glucopyranosyl-(1→2)-[*β*-*D*- xylopyranosyl -(1→3)]-*β*-*D*-glucopyranosyl(1→4)-*β*-*D*-galactopyranoside(japonicoside C).

Reference

1.Liu X, Zhang H, Niu X F, et al. Steroidal saponins from Smilacina japonica. Fitoterapia, 2012, 83(4):812-816.

S1. The IR spectrum of compound 1 (in KBr)

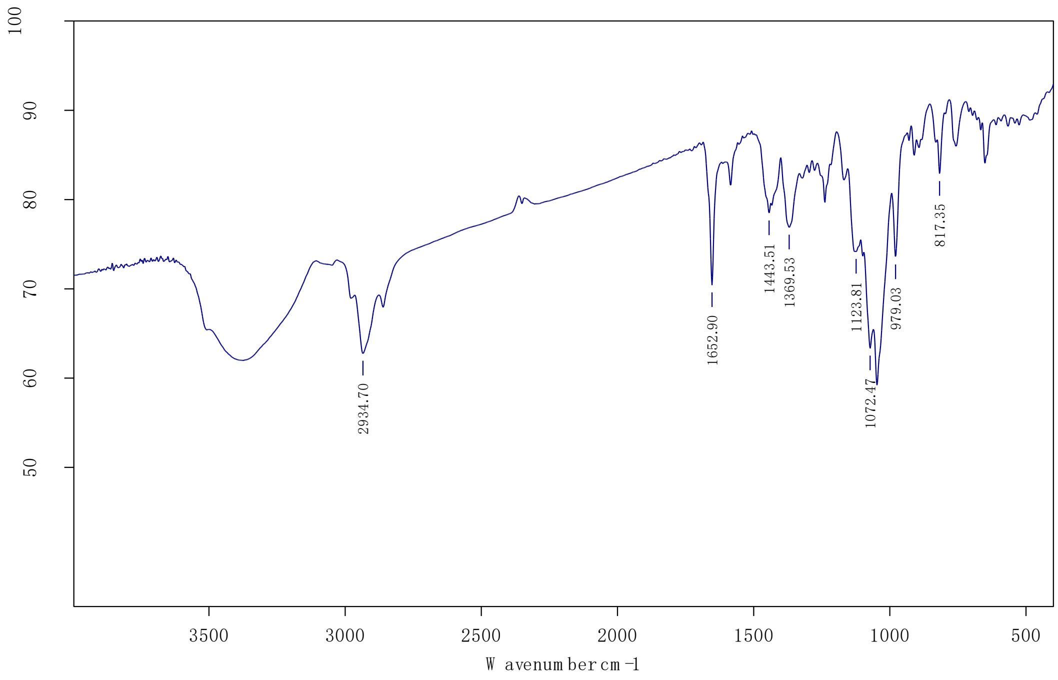

E :\ZD D \化合物2.2 Sam ple description Instrum ent type and / or accessory	2014-12-16

S2. The HR-ESI-MS spectrum of compound 1 (in MeOH)

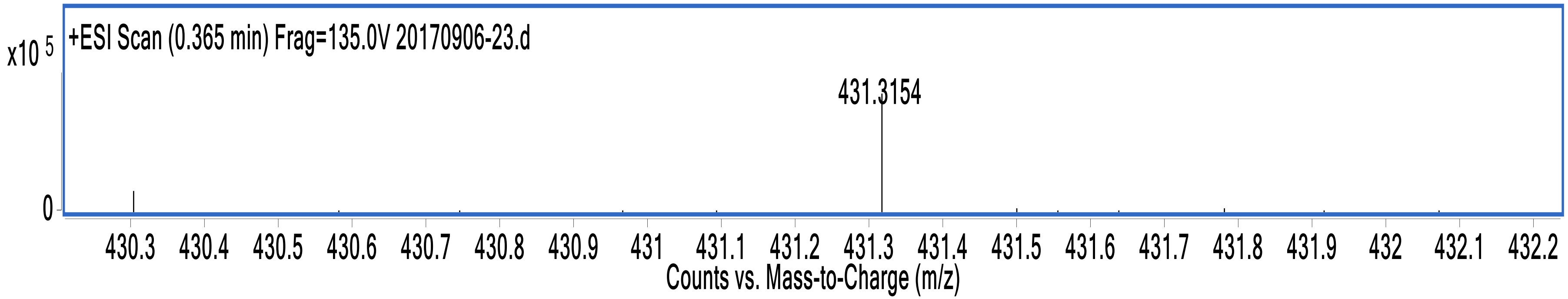

S3. The ^{1}H NMR spectrum of compound 1 (in pyridine-d5)

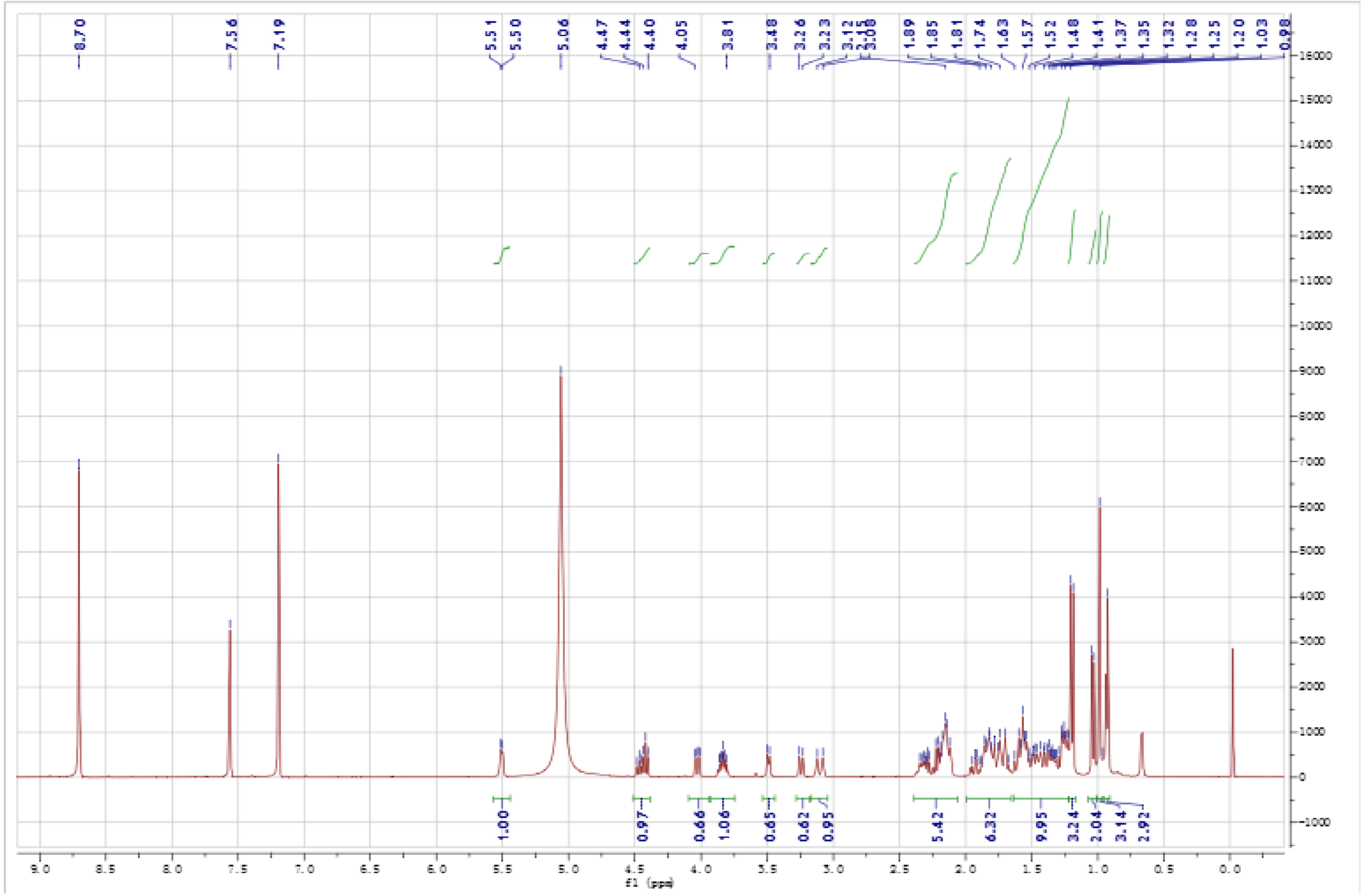

S4. The ^{13}C NMR spectrum of compound 1 (in pyridine-d5)

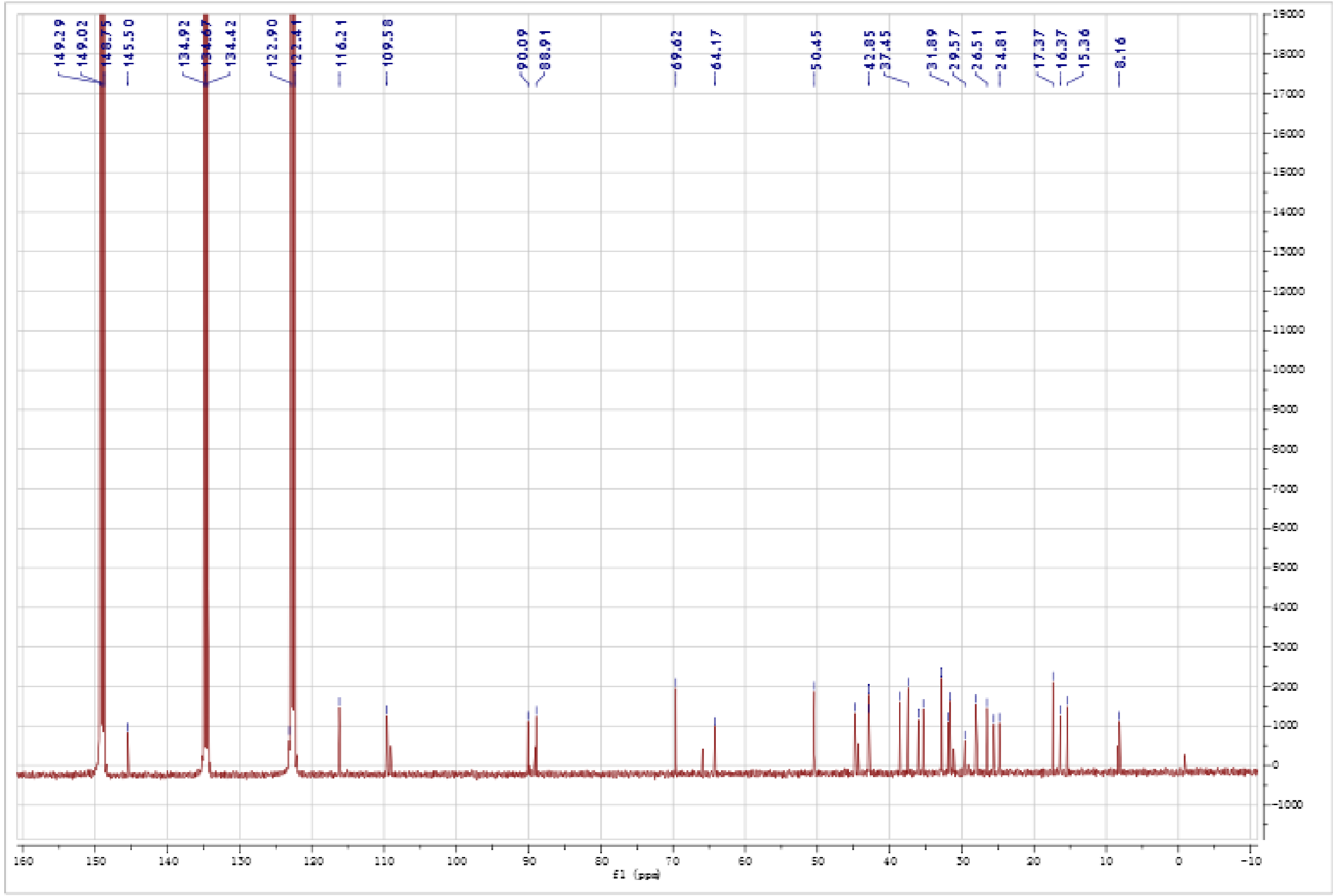

S5. The HSQC spectrum of compound 1 (in pyridine-d5)

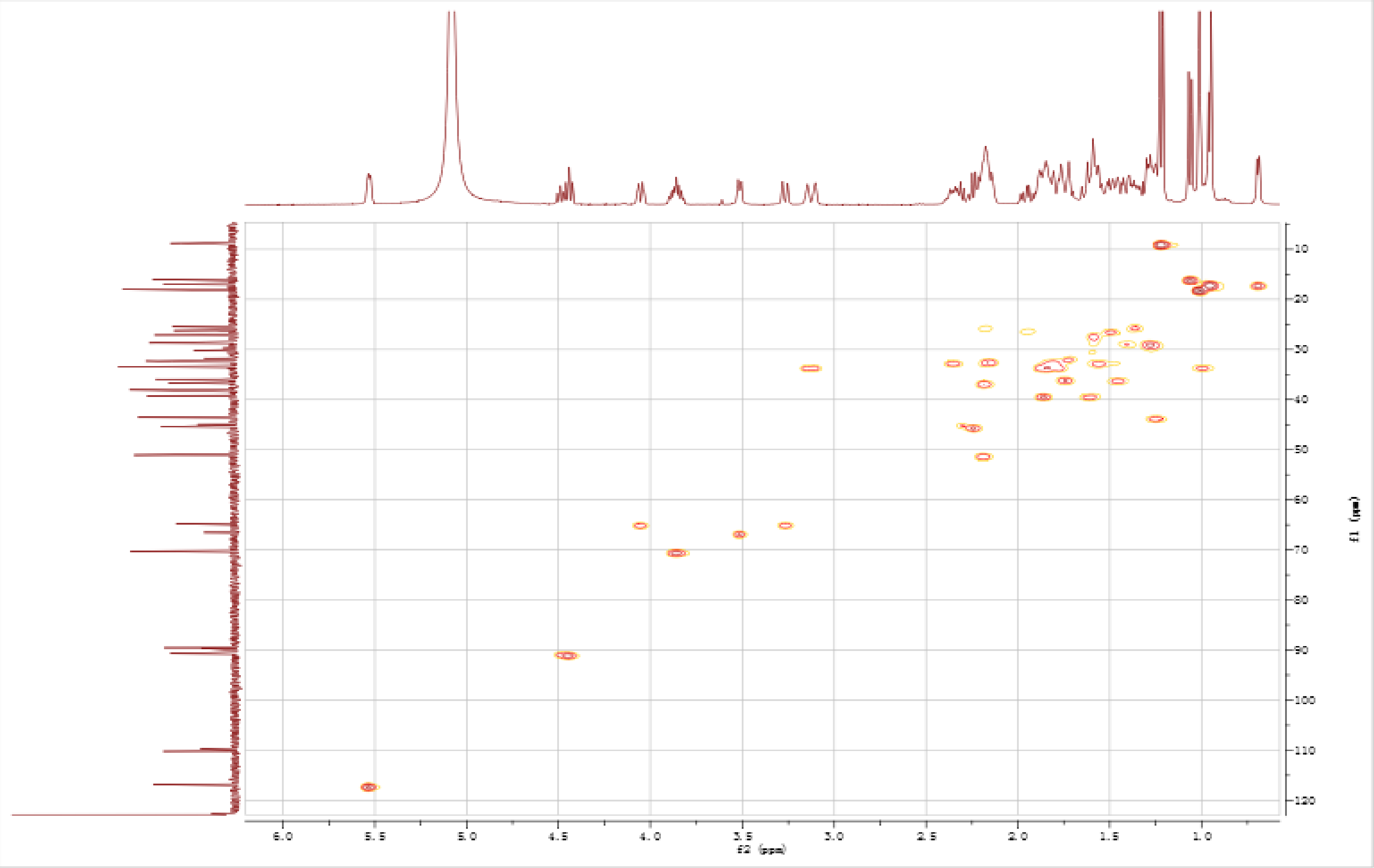

S6. The HMBC spectrum of compound 1 (in pyridine-d5)

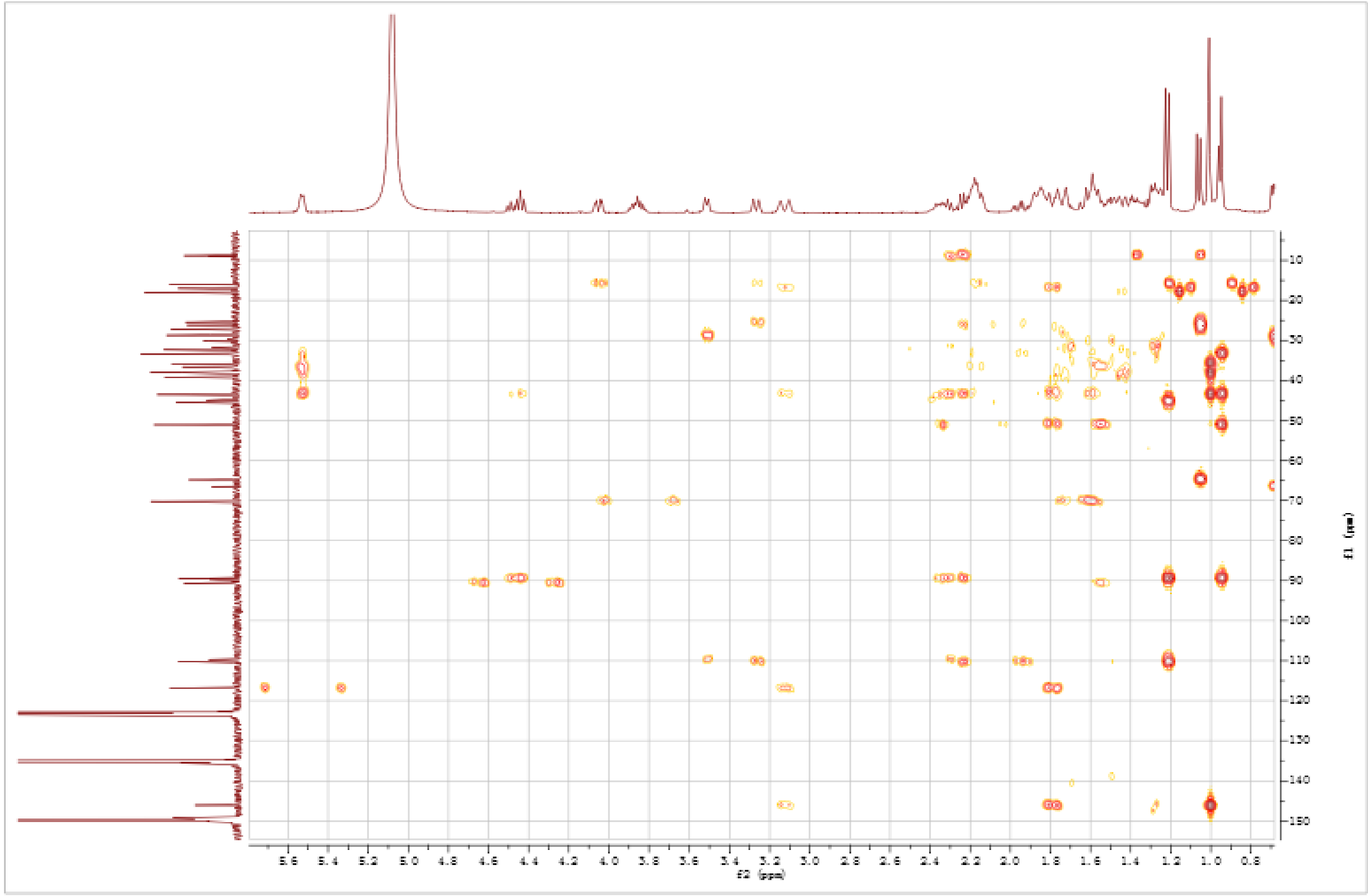

S7. The NOESY spectrum of compound 1(in pyridine-d5)

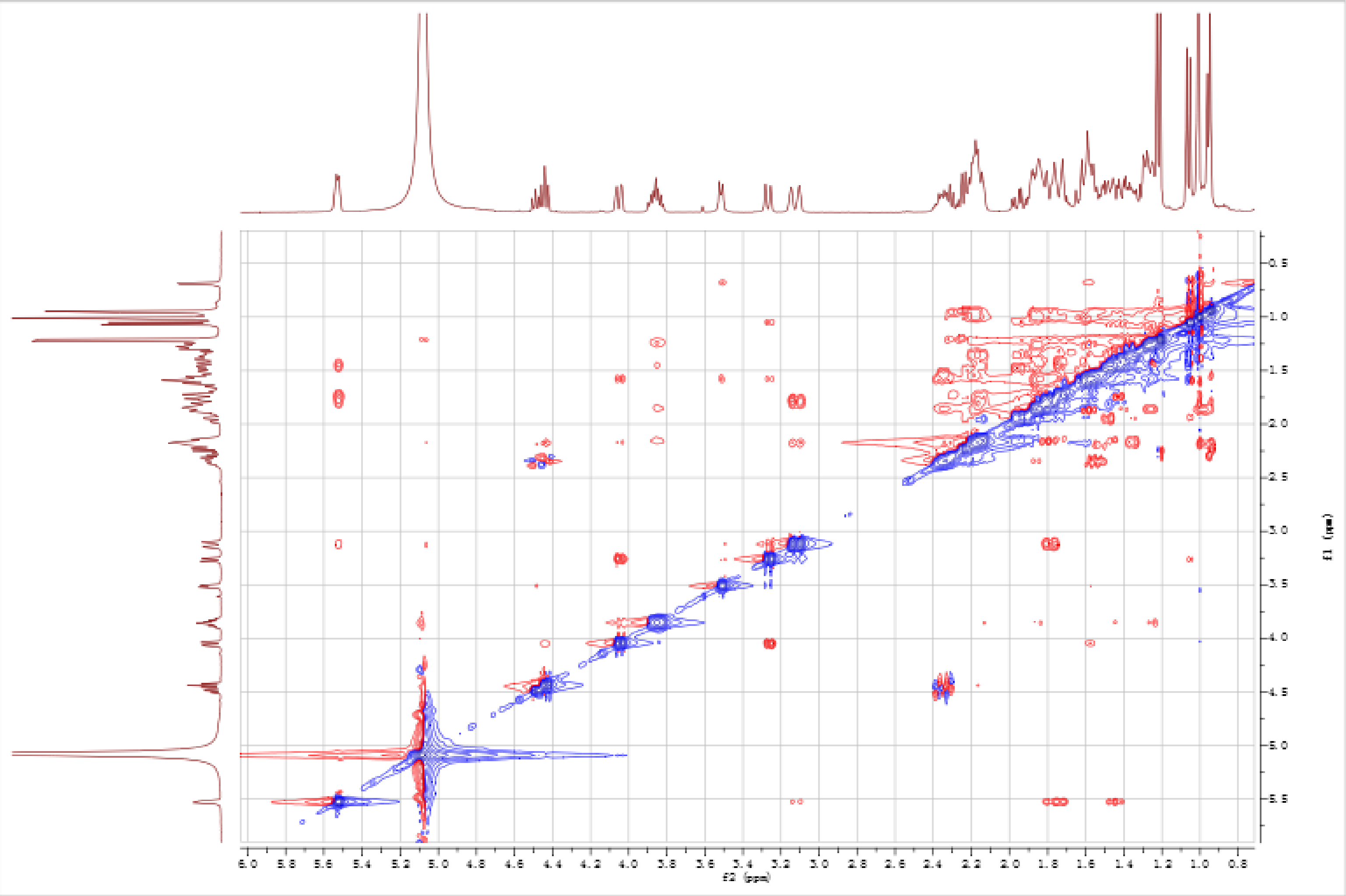

S8. The IR spectrum of compound 2 (in KBr)

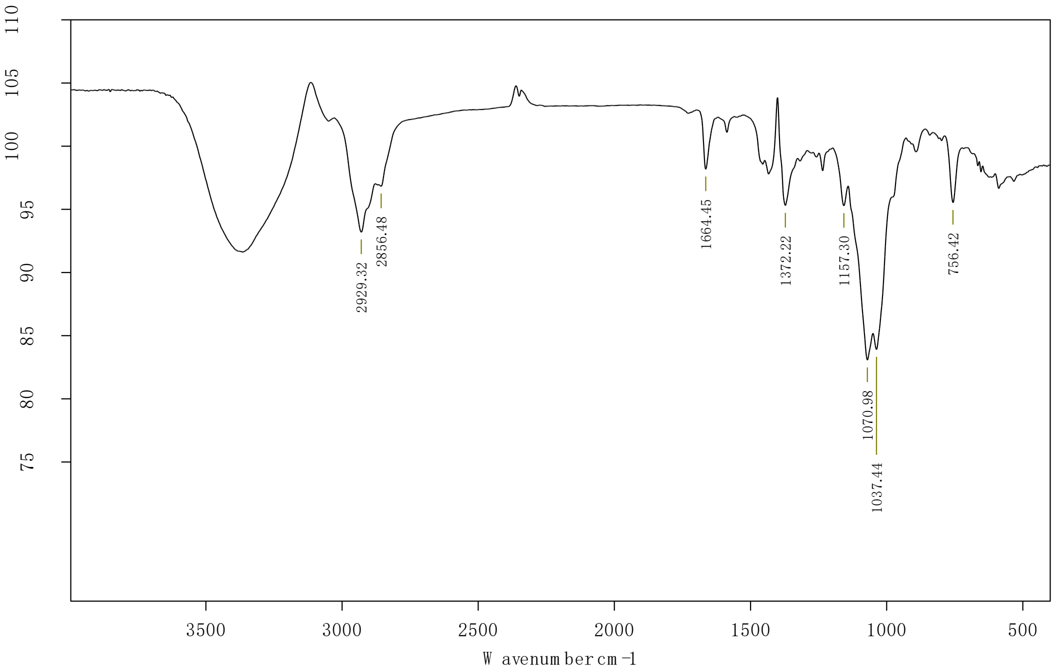

E:\ZDD\化合物1.2	Sam ple description	Instrum ent type and / or accessory	2014-12-16

S9. The HR-ESI-MS spectrum of compound 2 (in MeOH)

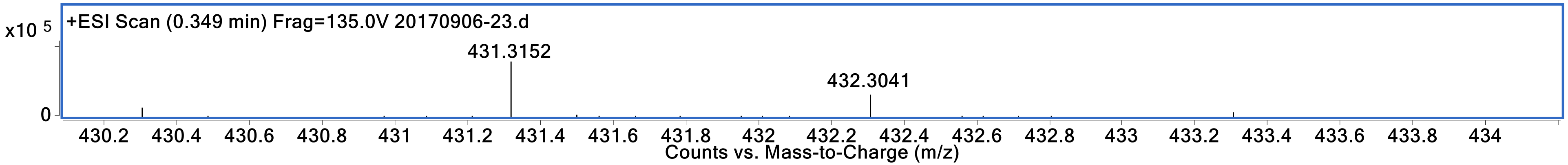

S10. The ¹H NMR spectrum of compound 2 (in pyridine-d

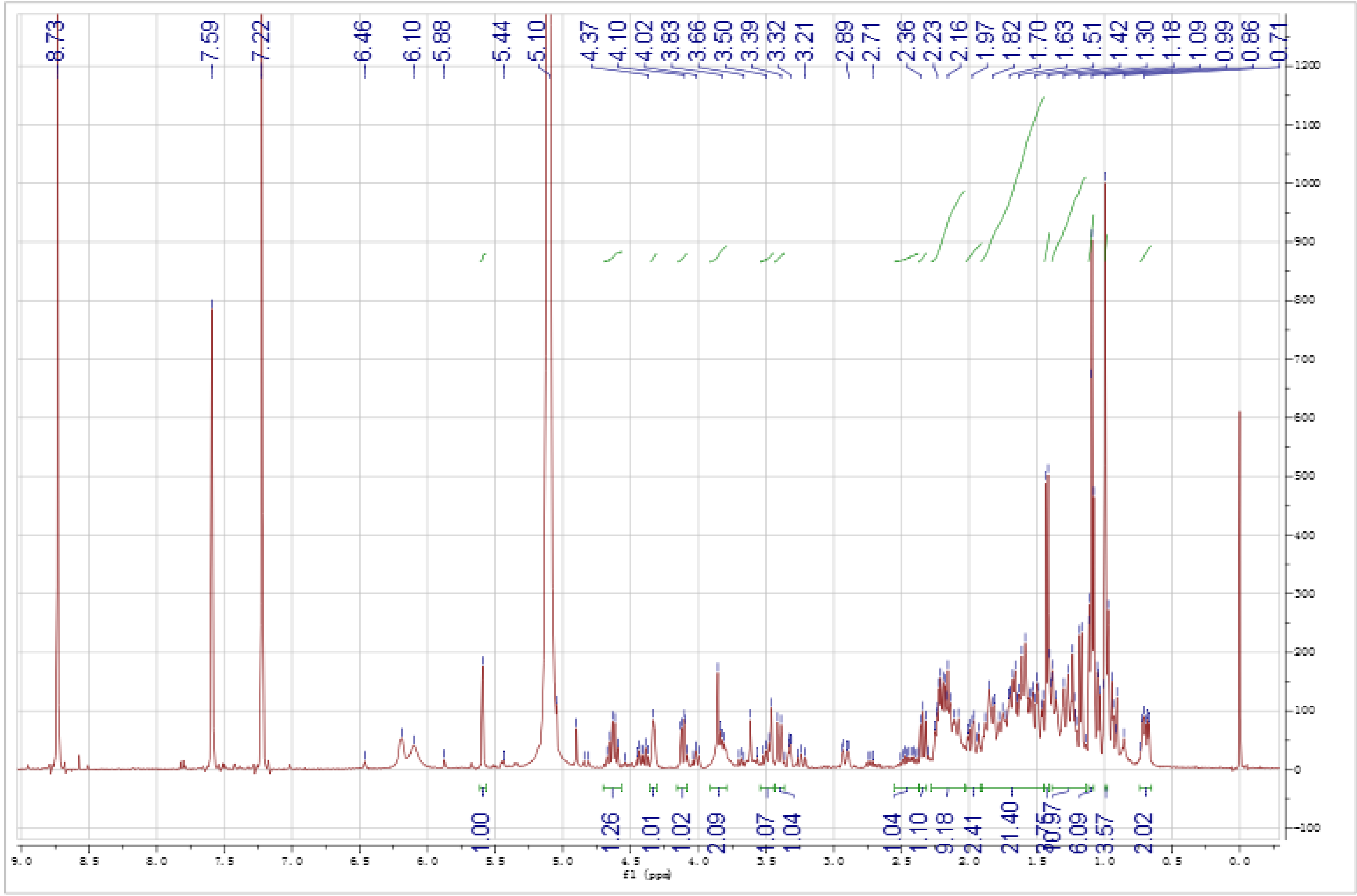

S11. The ^{13}C NMR spectrum of compound 2 (in pyridine-d5)

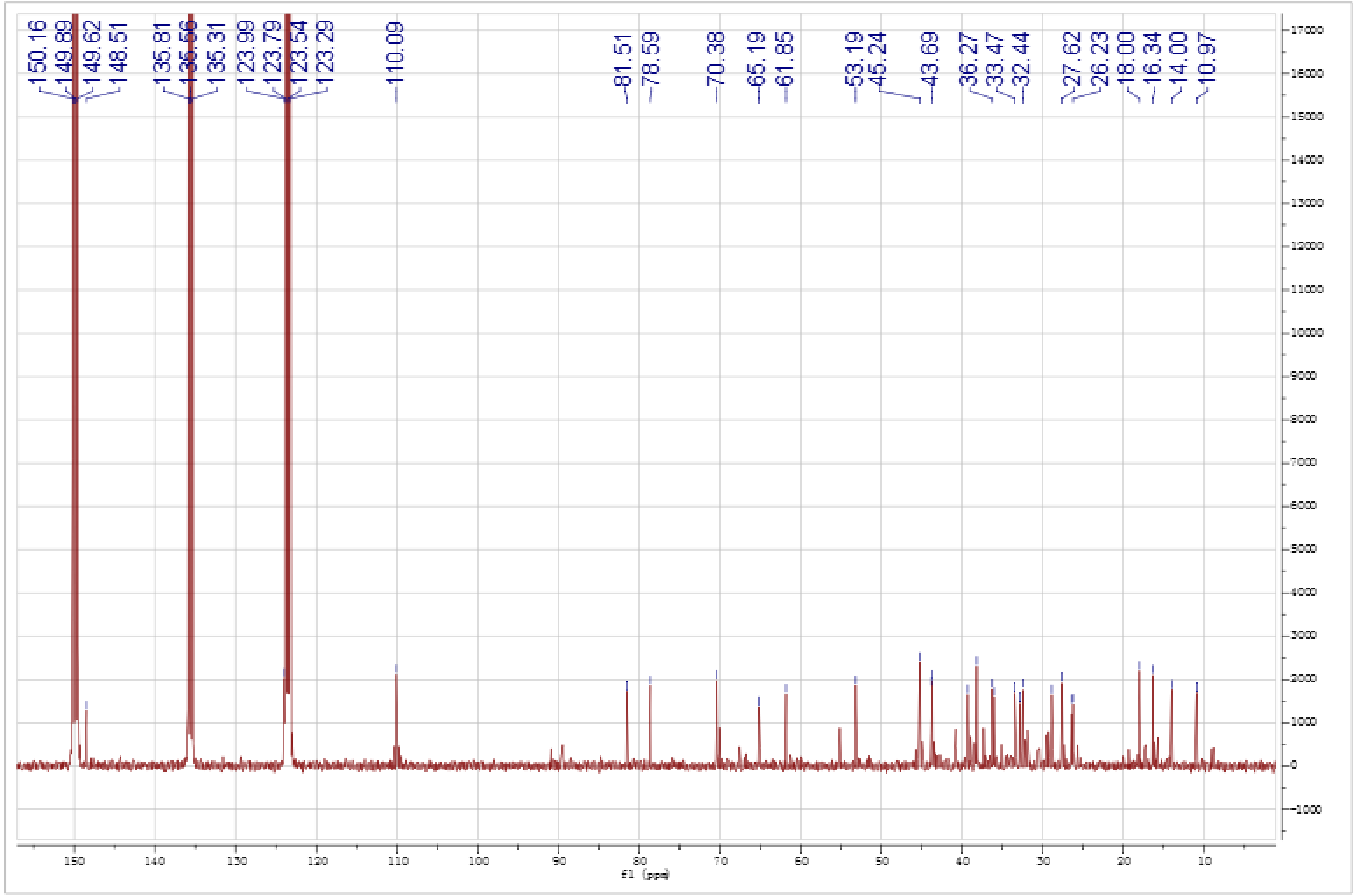

S12. The HSQC spectrum of compound 2 (in pyridine-d5)

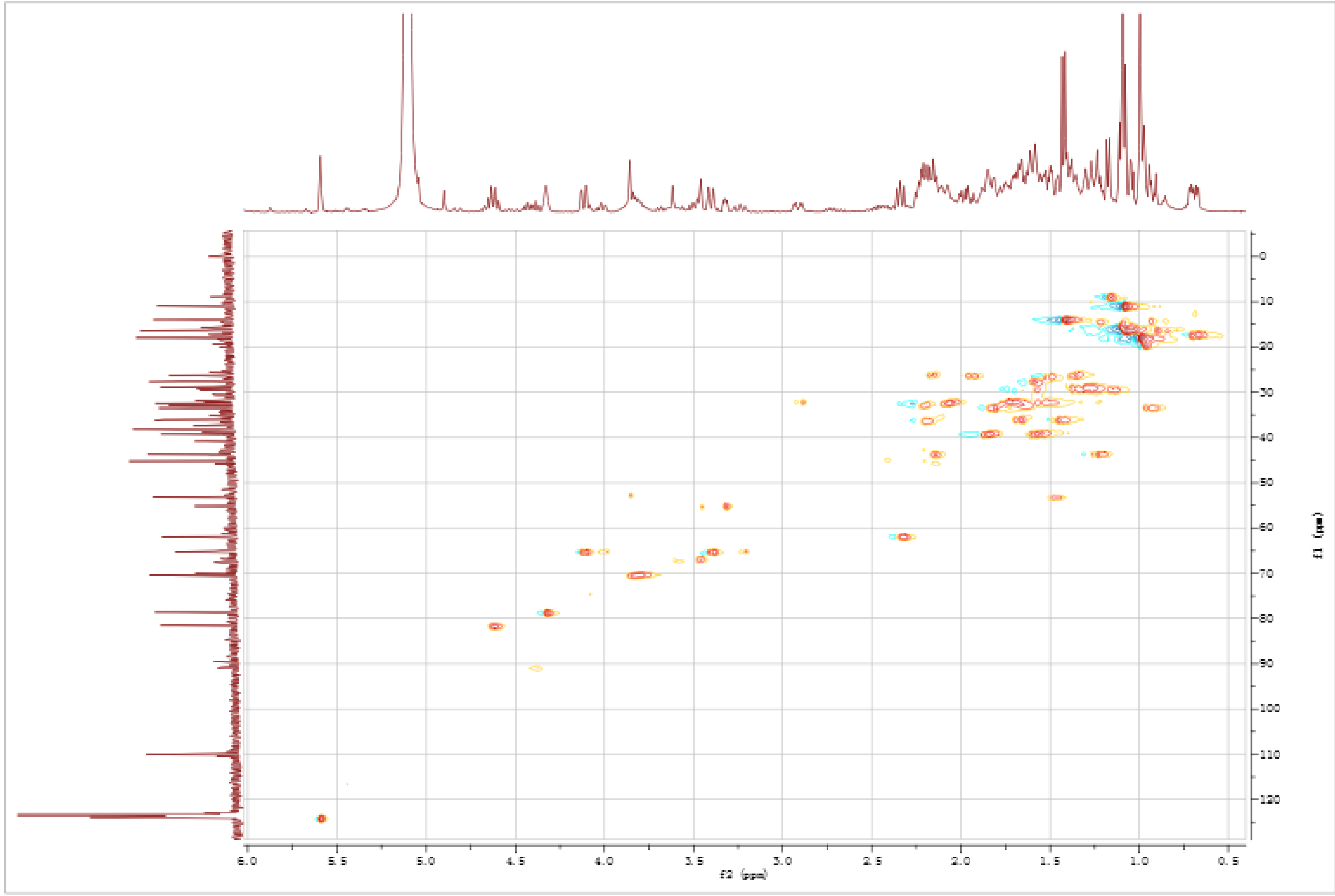

S13. The HMBC spectrum of compound 2 (in pyridine-d5)

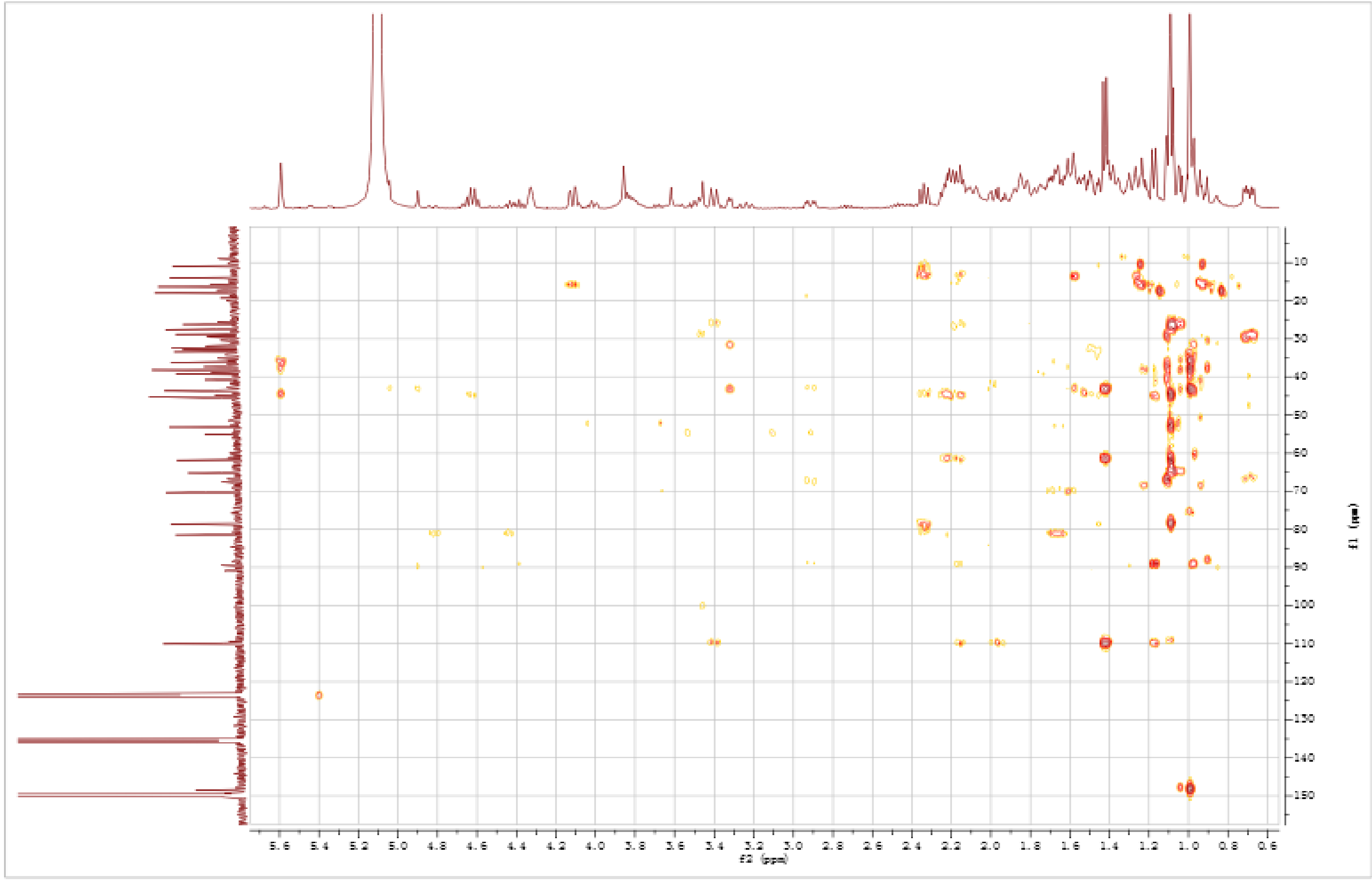

S14. The NOESY spectrum of compound 2 (in pyridine-d5)

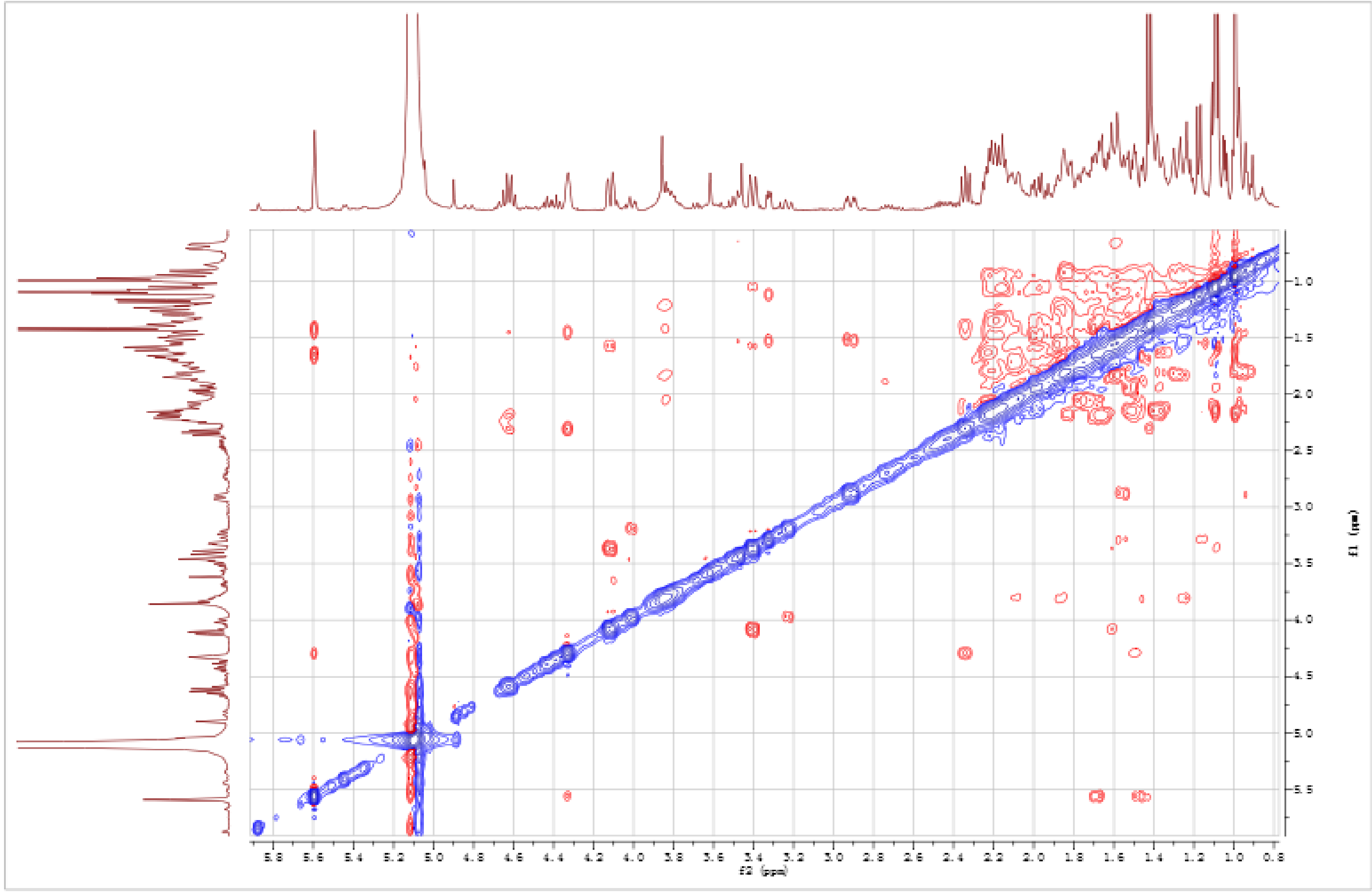

S15. The IR spectrum of compound 3 (in KBr)

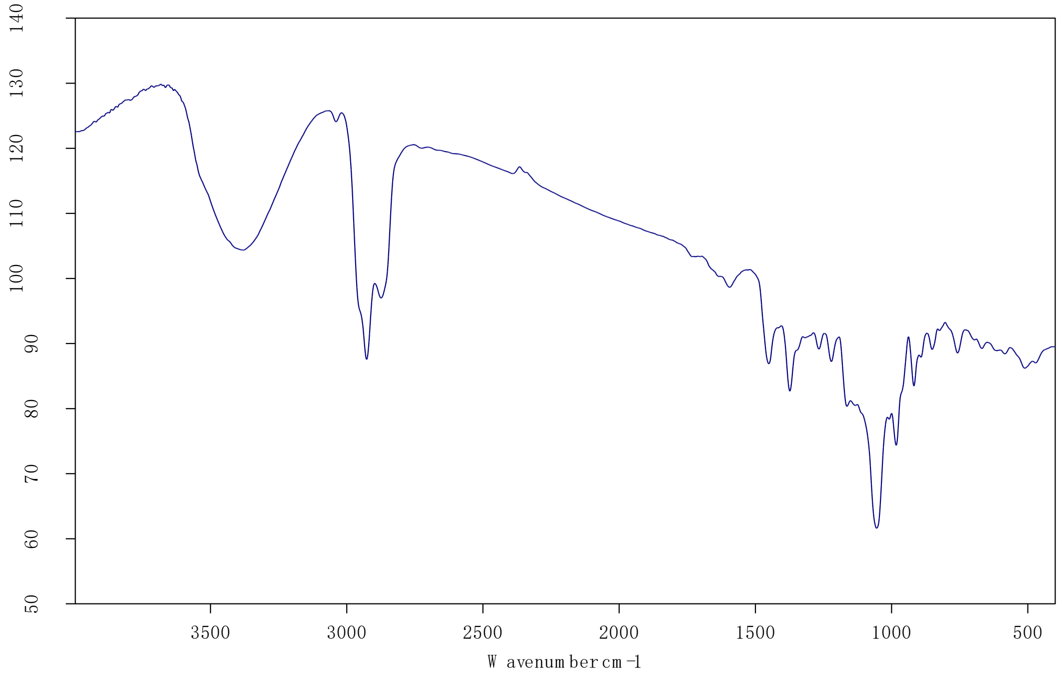

E :\ZDD \ly-1.2 物理混合物20170801 Instrum ent type and / or accessory	2017-8-4

S16. The HR-ESI-MS spectrum of compound 3 (in MeOH)

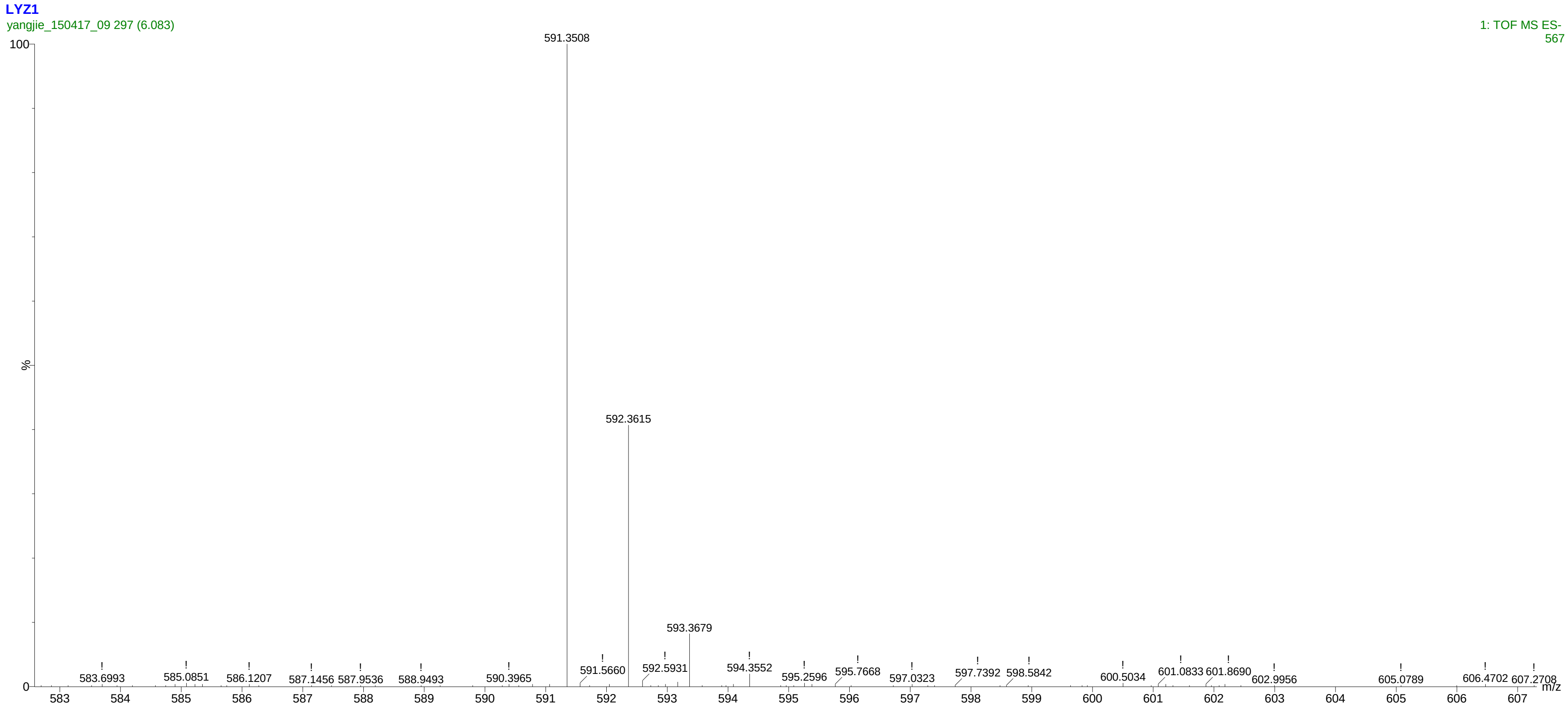

S17. The ^{1}H NMR spectrum of compound 3 (in pyridine-d5)

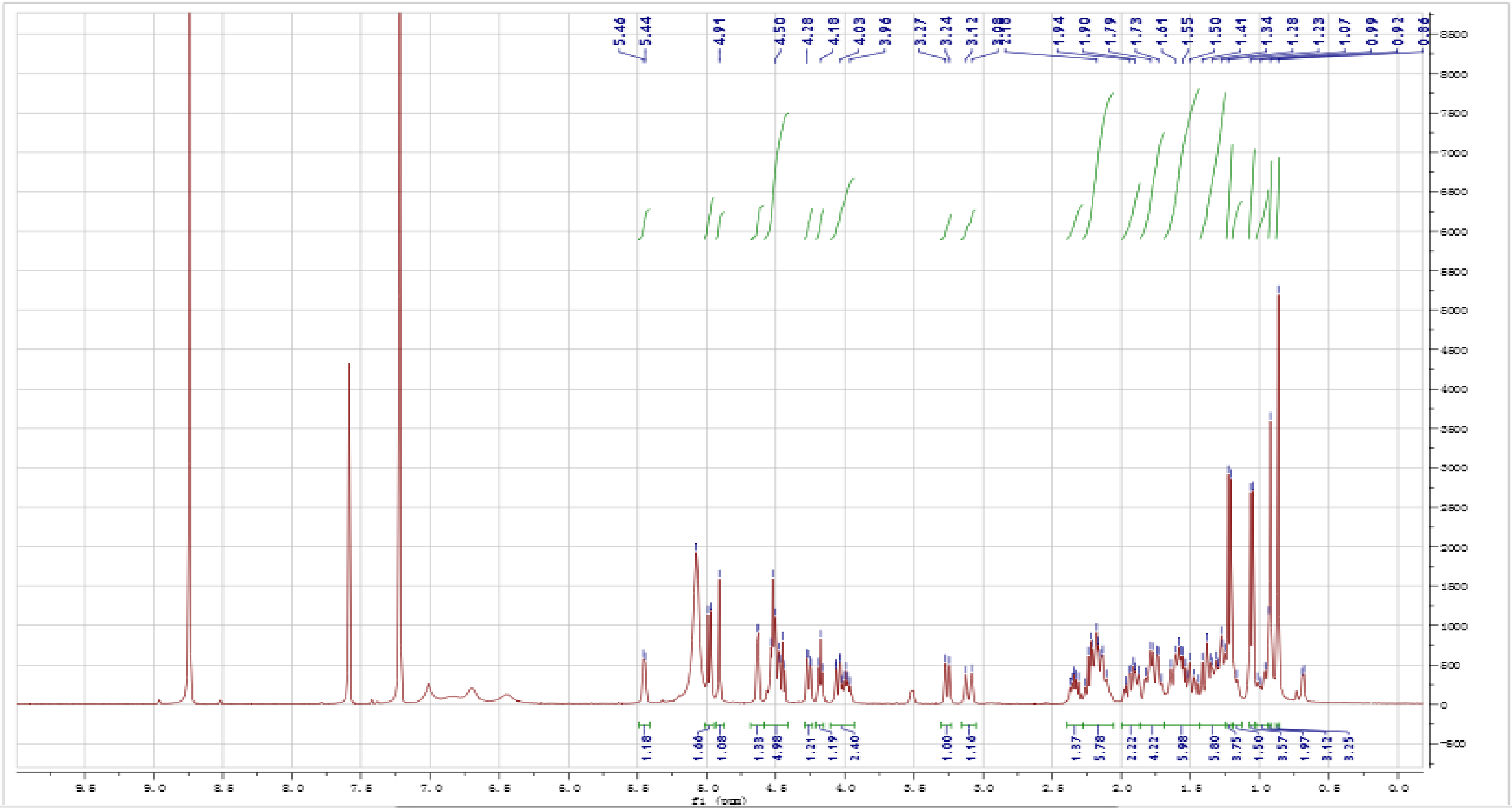

S18. The ^{13}C NMR spectrum of compound 3 (in pyridine-d5)

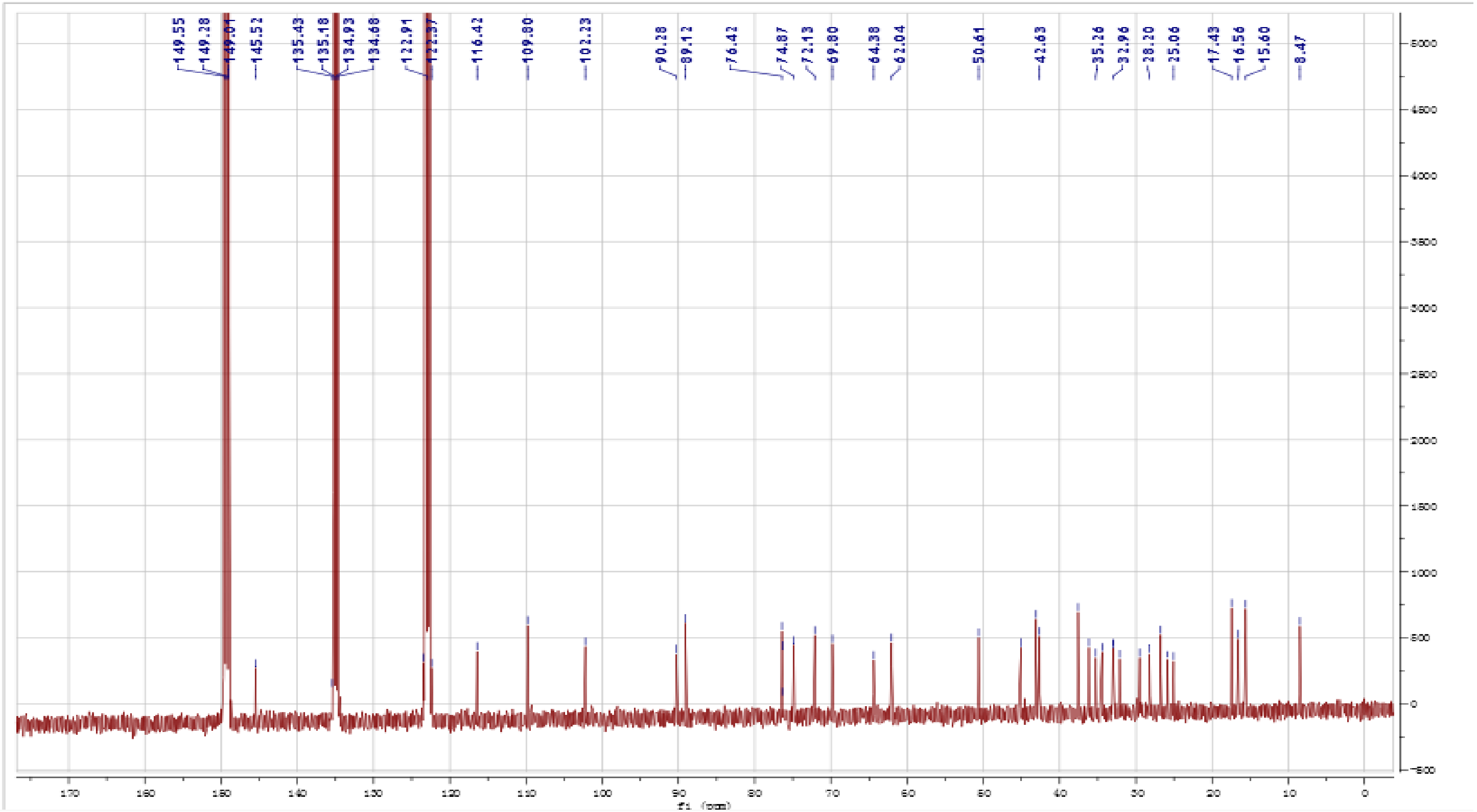

S19. The HSQC spectrum of compound 3 (in pyridine-d5)

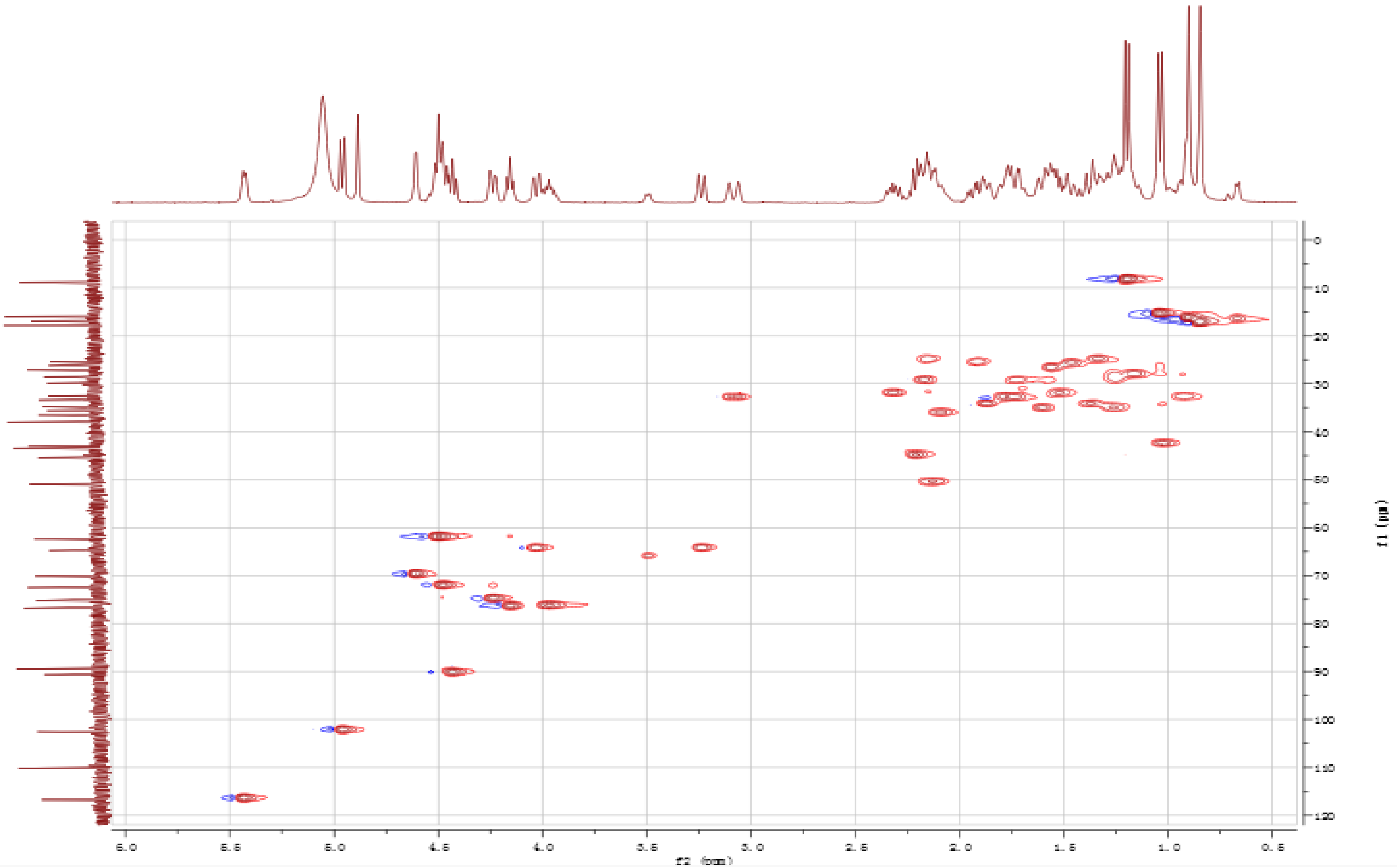

S20. The HMBC spectrum of compound 3 (in pyridine-d5)

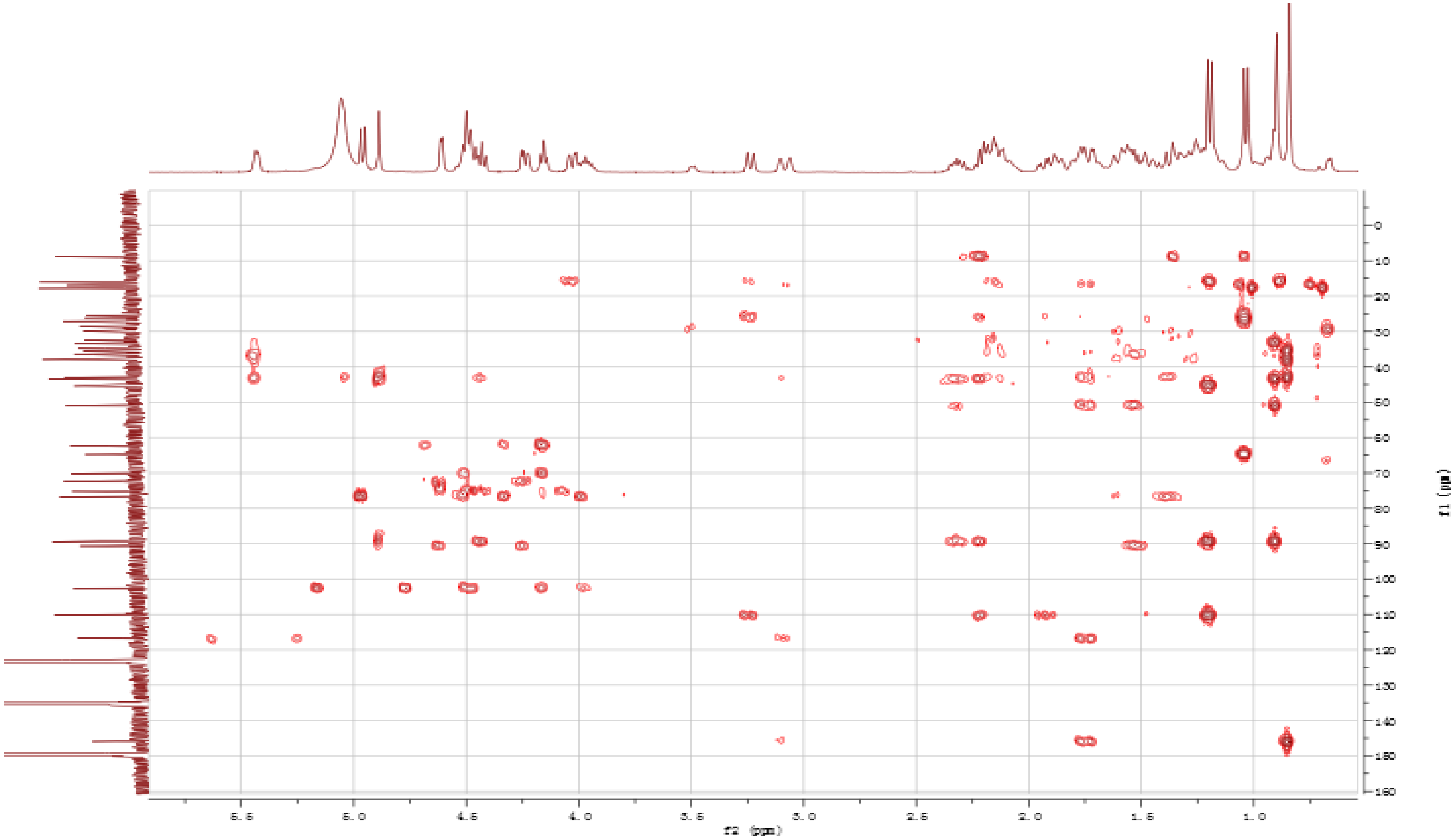

S21. The NOESY spectrum of compound 3 (in pyridine-d5)

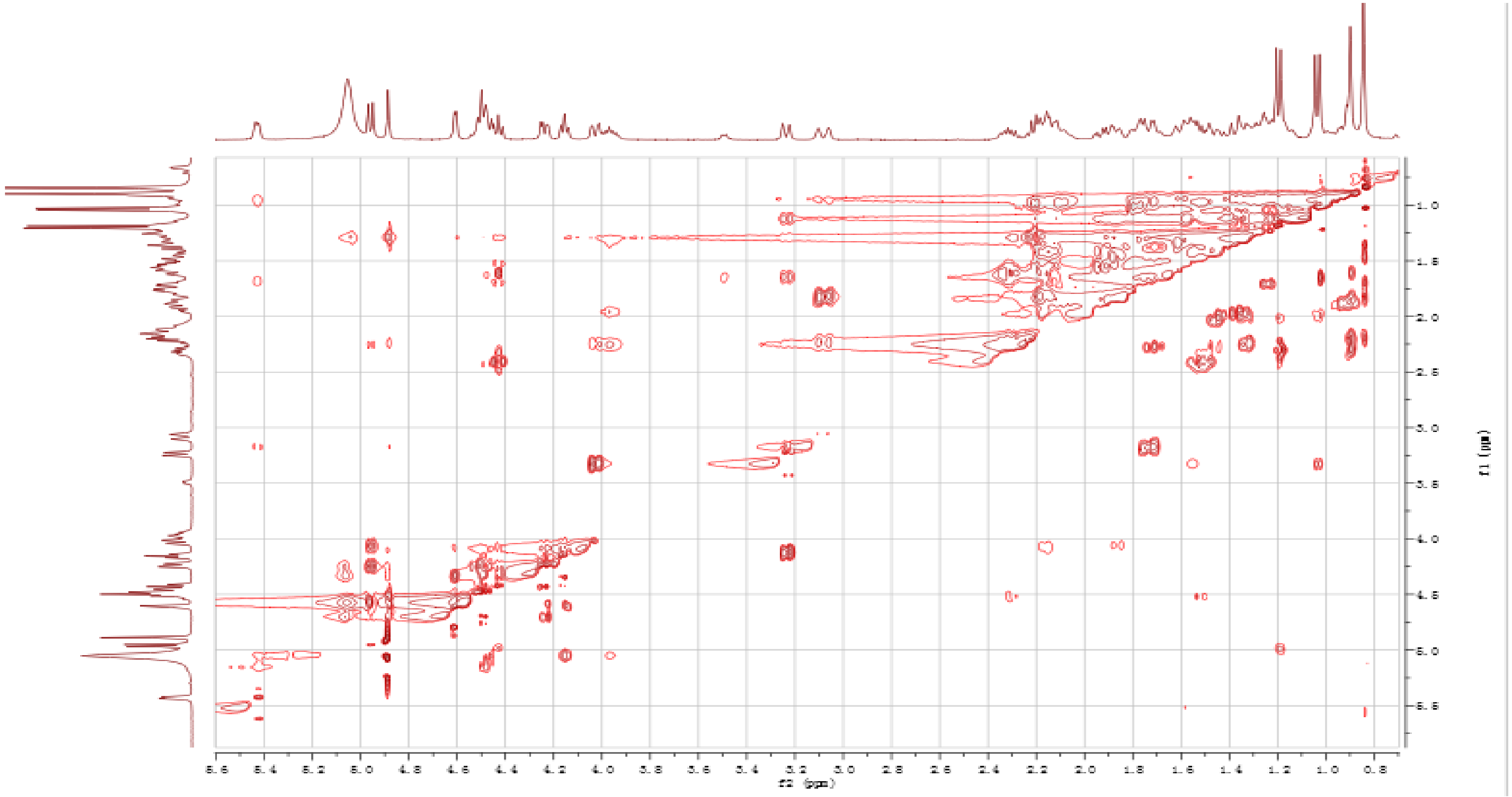

S22. The IR spectrum of compound 4 (in KBr)

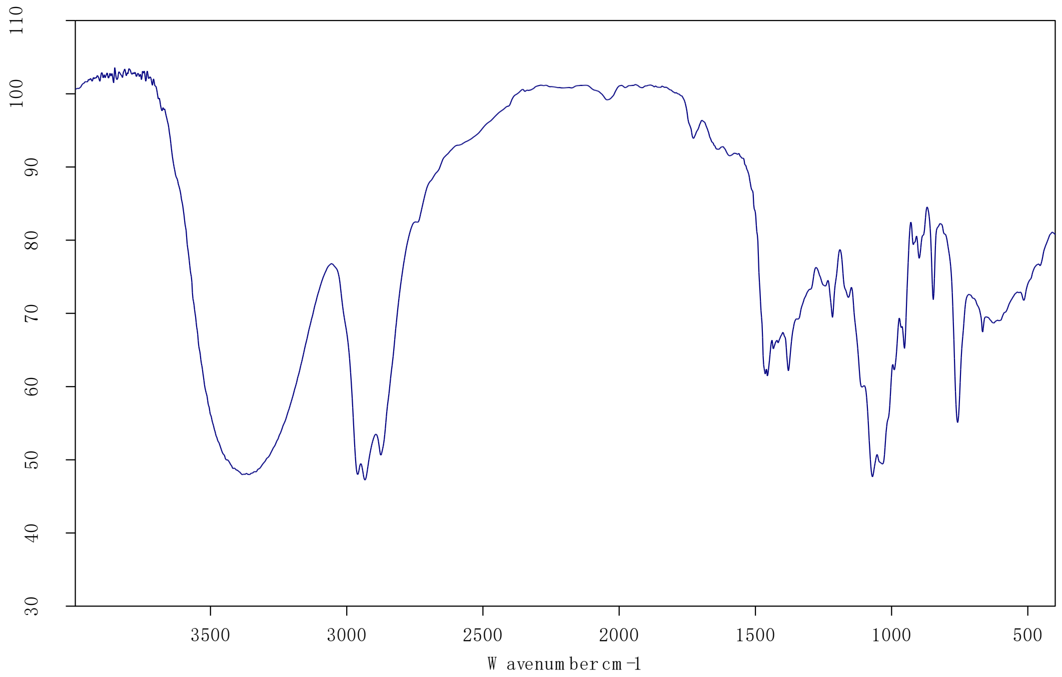

E :\ZDD \ly-2.0 物理混合物20170801 Instrum ent type and / or accessory	2017-8-4

S23. The HR-ESI-MS spectrum of compound 4 (in MeOH)

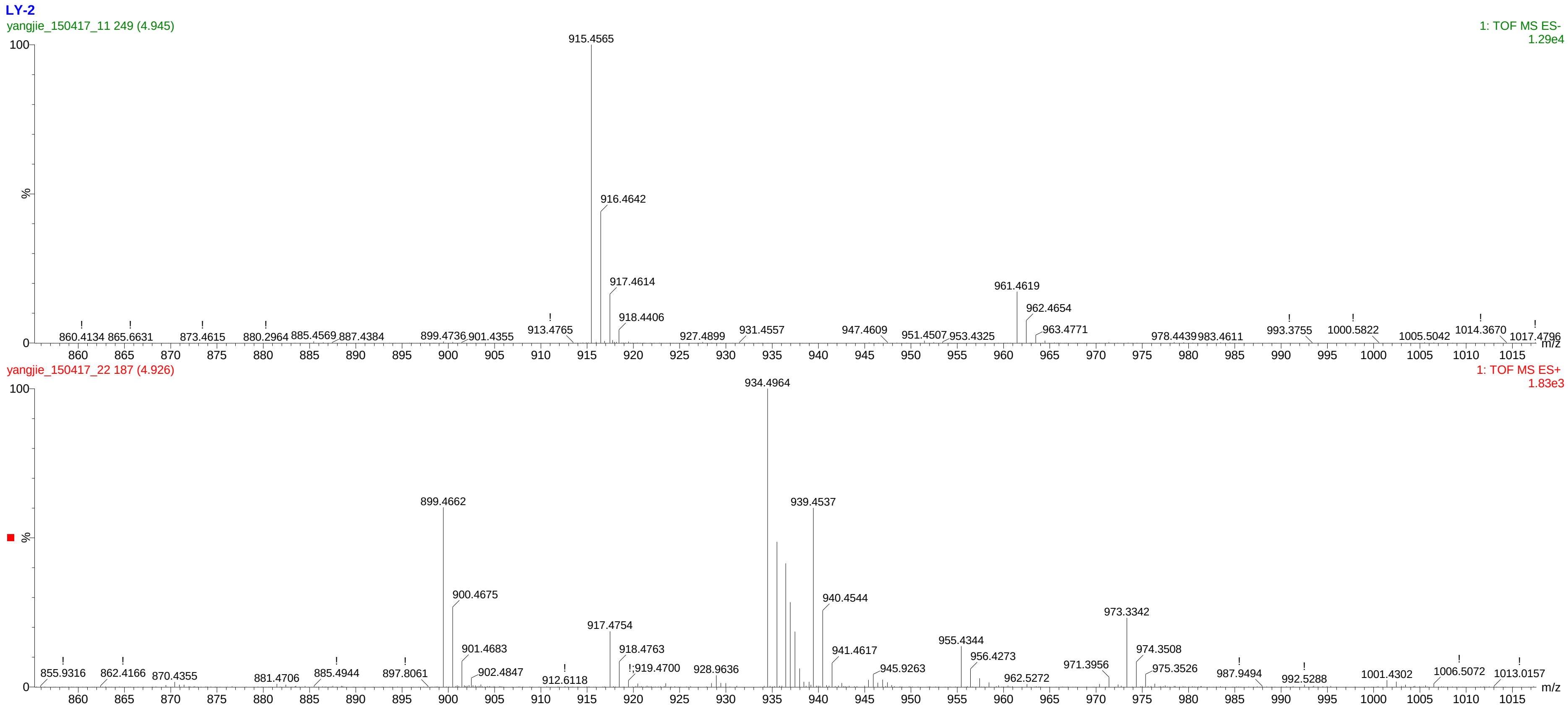

S24. The ^{1}H NMR spectrum of compound 4(in pyridine-d5)

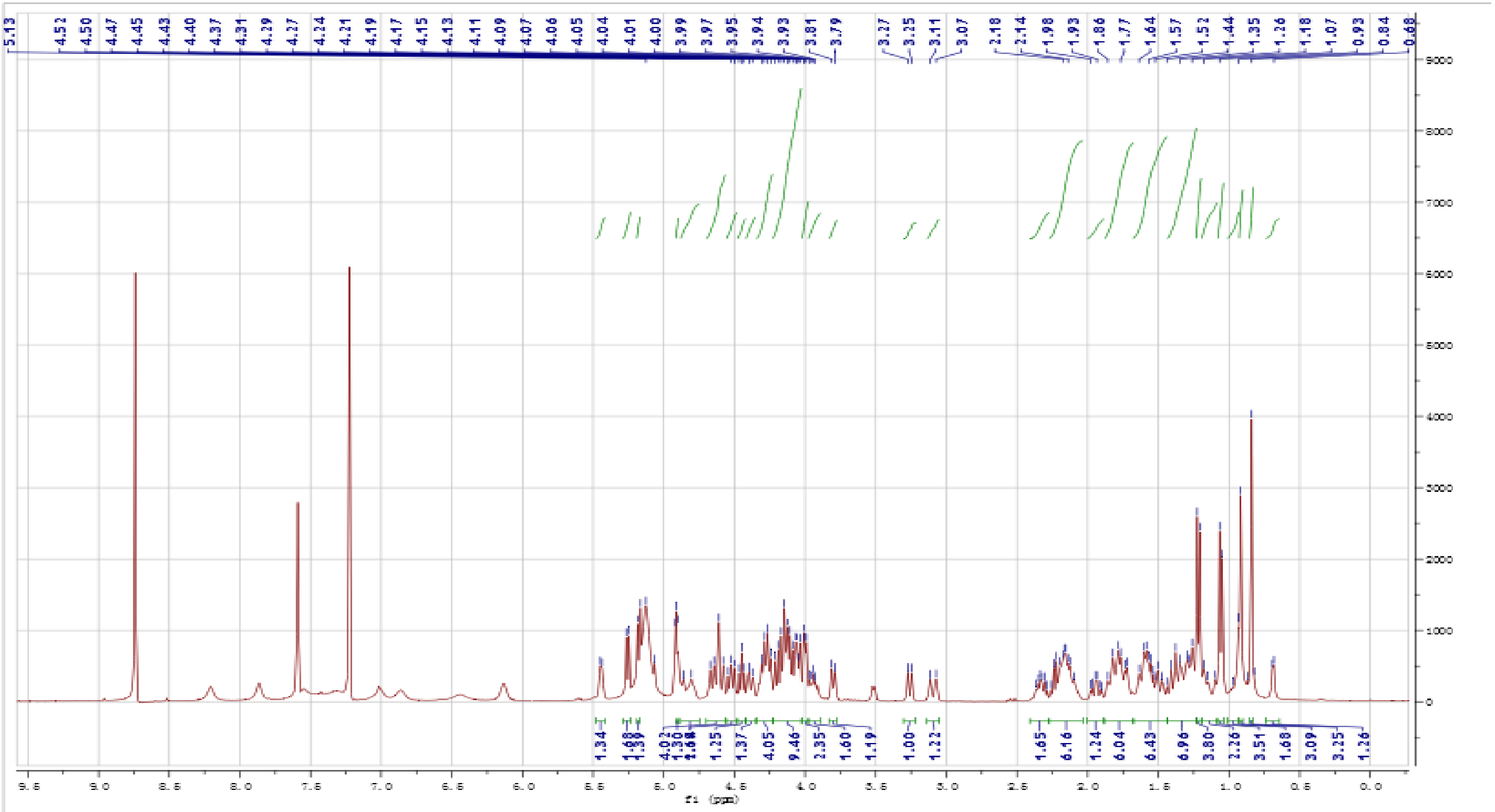

S25. The ^{13}C NMR spectrum of compound 4 (in pyridine-d5)

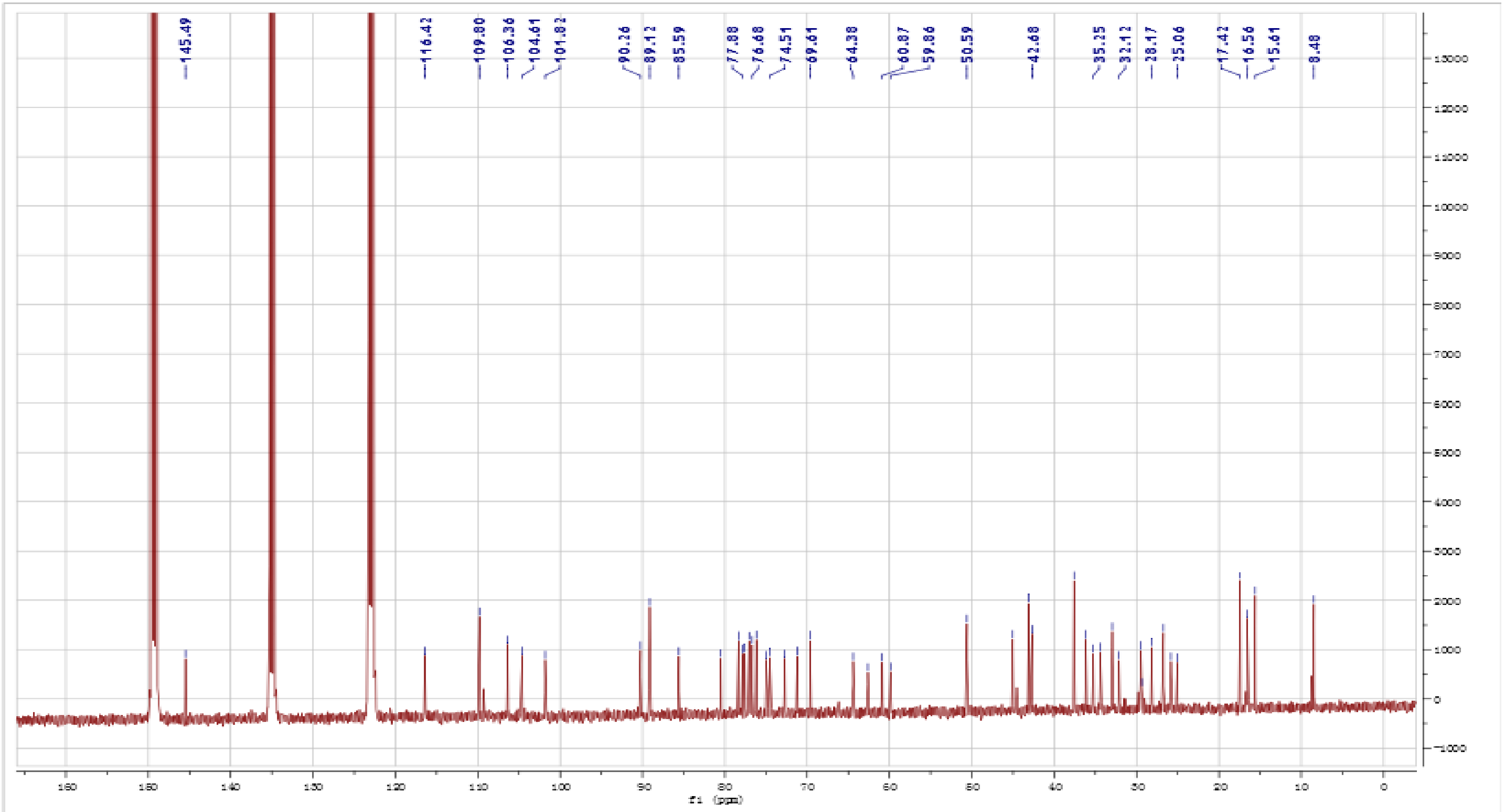

S26. The HSQC spectrum of compound 4 (in pyridine-d5)

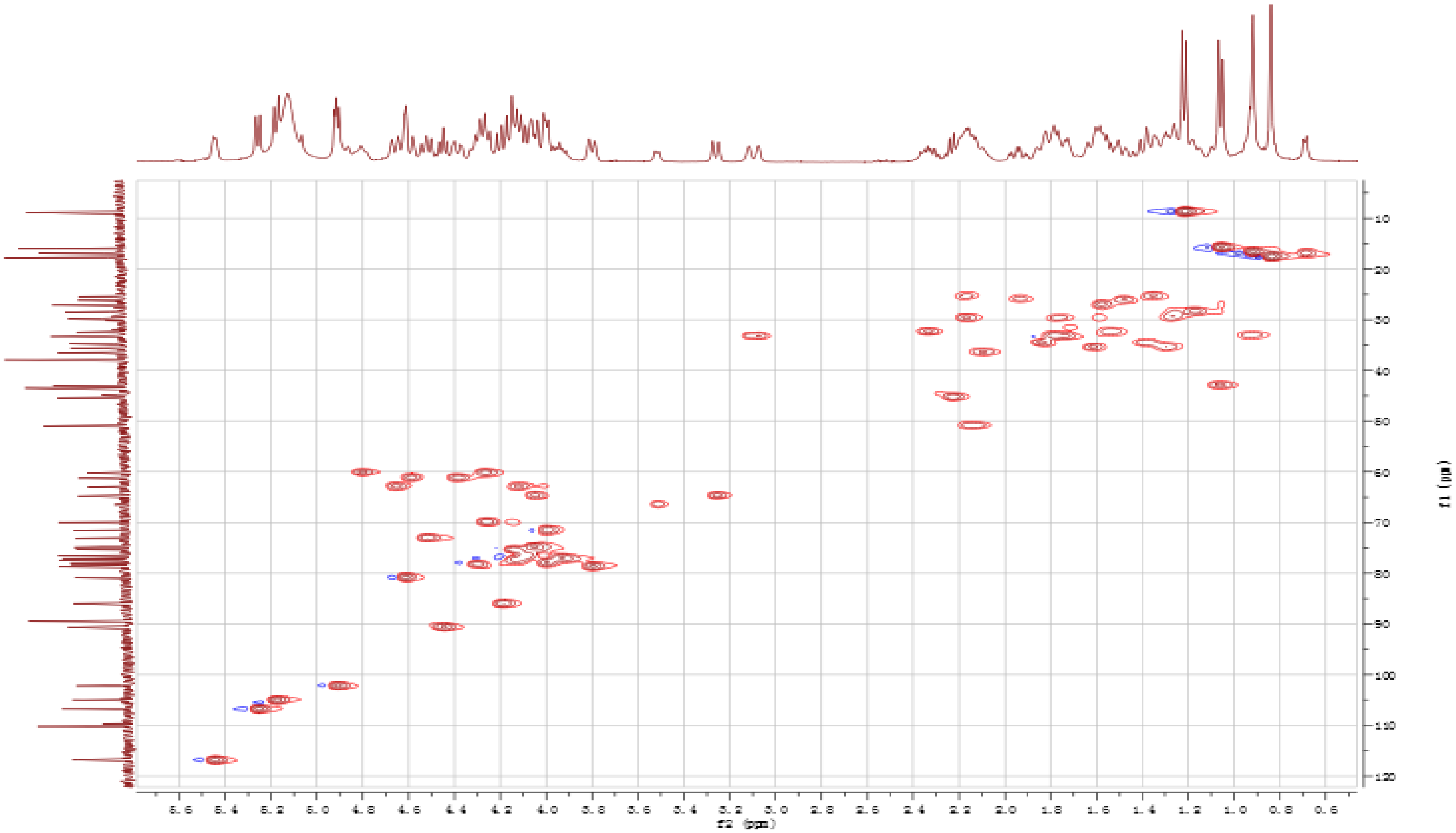

S27. The HMBC spectrum of compound 4 (in pyridine-d5)

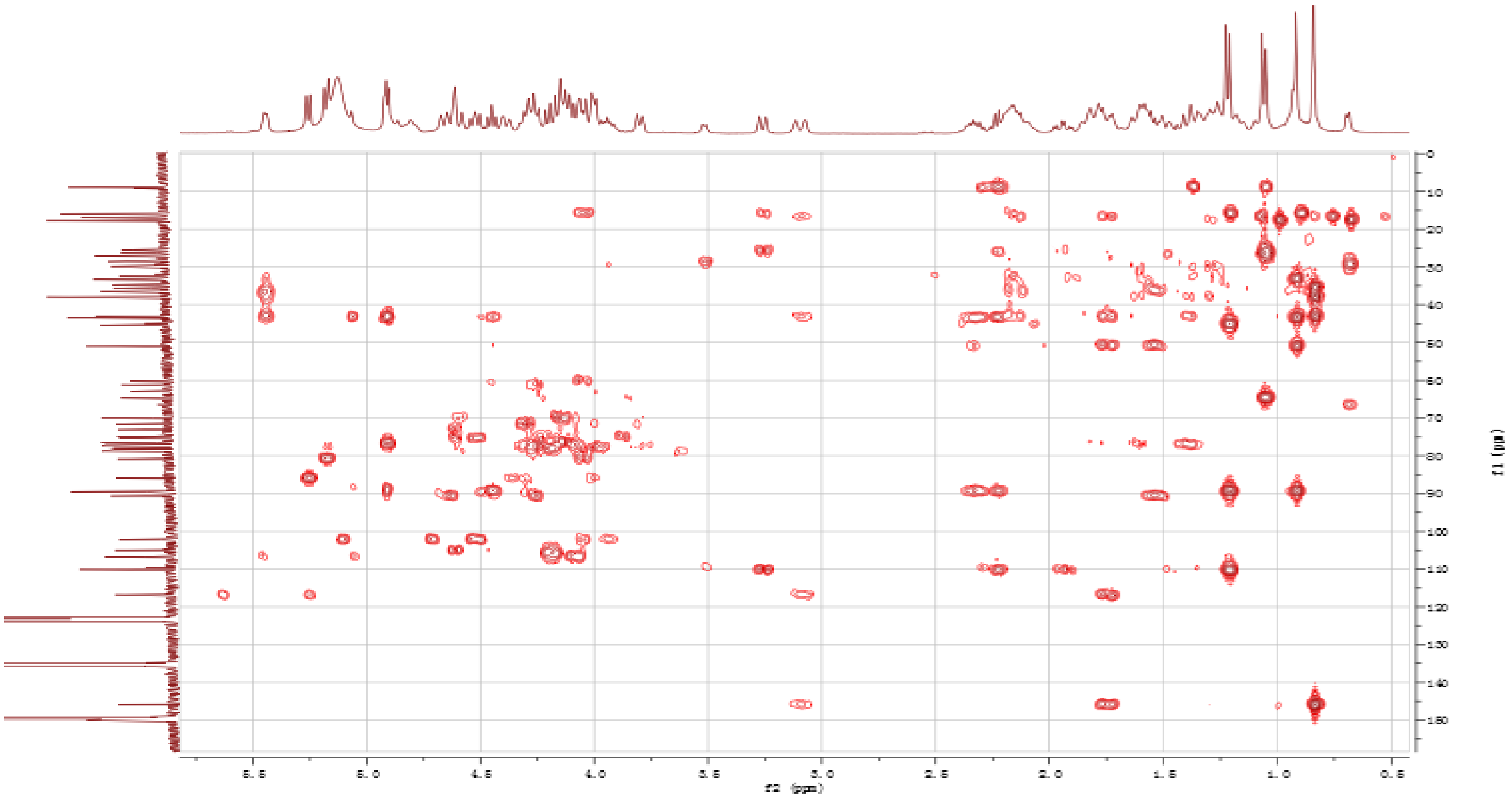

S28. The NOESY spectrum of compound 4 (in pyridine-d5)

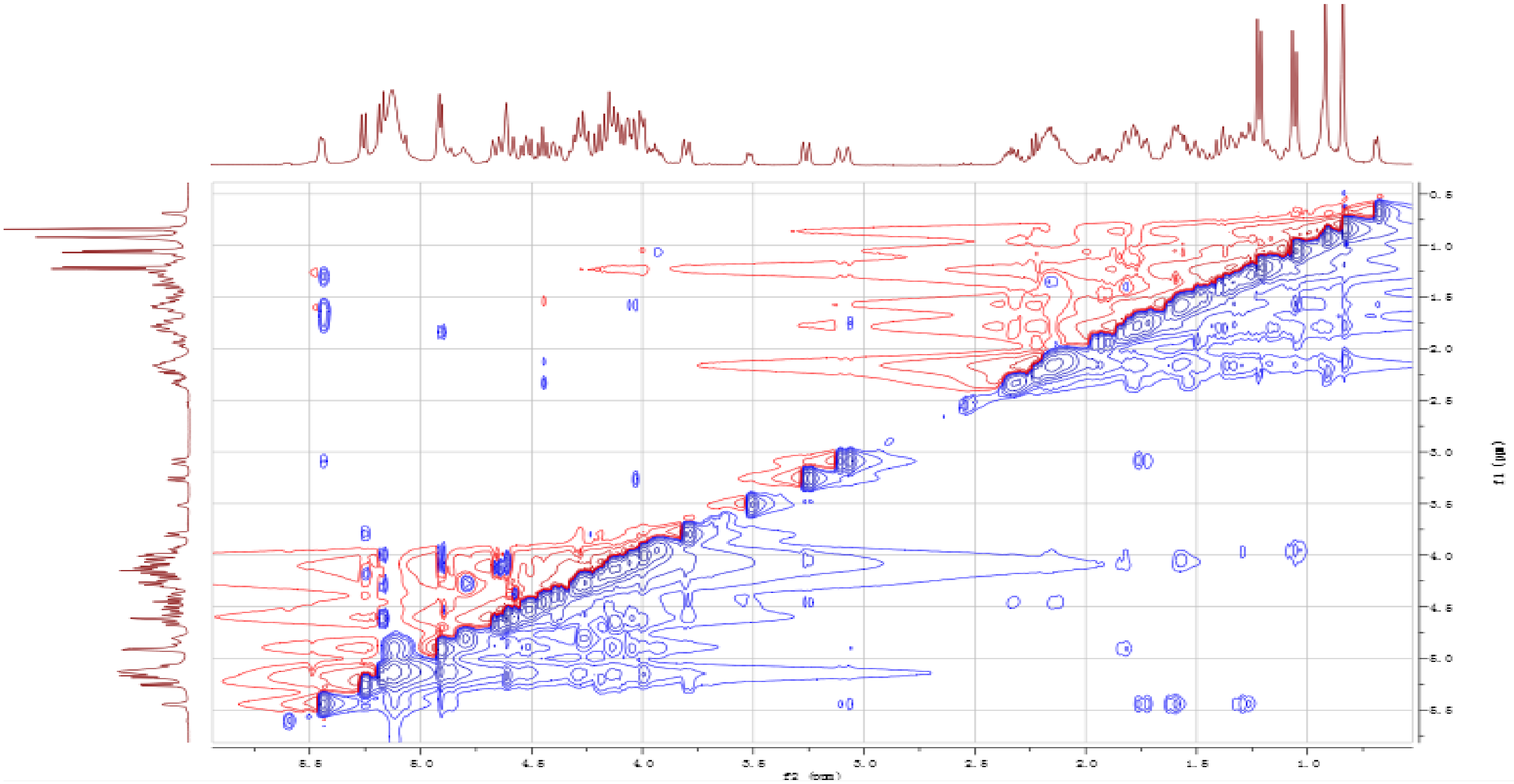

Supplement: Supplementary file 1 [file molecules-23-00798-s001.pdf]
